# GOTA: GO term annotation of biomedical literature

**DOI:** 10.1186/s12859-015-0777-8

**Published:** 2015-10-28

**Authors:** Pietro Di Lena, Giacomo Domeniconi, Luciano Margara, Gianluca Moro

**Affiliations:** Department of Computer Science and Engineering, University of Bologna, Cesena Campus, Via Sacchi 3, Cesena, 47521 Italy

**Keywords:** Automated annotation, Text mining, Gene Ontology

## Abstract

**Background:**

Functional annotation of genes and gene products is a major challenge in the post-genomic era. Nowadays, gene function curation is largely based on manual assignment of Gene Ontology (GO) annotations to genes by using published literature. The annotation task is extremely time-consuming, therefore there is an increasing interest in automated tools that can assist human experts.

**Results:**

Here we introduce GOTA, a GO term annotator for biomedical literature. The proposed approach makes use only of information that is readily available from public repositories and it is easily expandable to handle novel sources of information. We assess the classification capabilities of GOTA on a large benchmark set of publications. The overall performances are encouraging in comparison to the state of the art in multi-label classification over large taxonomies. Furthermore, the experimental tests provide some interesting insights into the potential improvement of automated annotation tools.

**Conclusions:**

GOTA implements a flexible and expandable model for GO annotation of biomedical literature. The current version of the GOTA tool is freely available at http://gota.apice.unibo.it.

**Electronic supplementary material:**

The online version of this article (doi:10.1186/s12859-015-0777-8) contains supplementary material, which is available to authorized users.

## Background

The Gene Ontology (GO) project [1] is a major collaborative initiative, started in 1998 with the aim to unify the representation of gene and gene product attributes across all species. Nowadays, GO is the de facto standard for functional annotation of genes [2, 3]. The two main efforts of the GO project involve: i) the development and maintenance of a controlled vocabulary (ontologies) of functional attributes; ii) the annotation of genes in terms of the their associated attributes.

Nowadays, the majority of GO annotations are assigned by using computational methods [4–8], although electronically inferred annotations are usually considered as inaccurate and unreliable [9, 10]. At the state of the art, GO annotations derived from manual curation of scientific literature can be still regarded as the *gold-standard* in terms of quality and specificity. However, the manual annotation step is extremely time-consuming, and thus it is one of the major bottlenecks in GO curation. The annotation task has become an even harder challenge in the post-genomic era, which has been characterized by an unprecedented growth in the production of biomedical literature. As an example, The Arabidopsis Information Resource’s curation team (TAIR) reports that, in the recent years, it has been able to curate only a relatively small fraction (∼30 %) of the newly published literature on *Arabidopsis thaliana* [11]. Due to this enormous growth of biological information in form of unstructured text, there is an increasing interest in text mining tools that can aid GO curators during the labor-intensive annotation task [12].

The main reference for the state-of-the-art in automated GO curation is the *text-mining challenge task for literature-based GO annotation* at the BioCreative experiments [13, 14]. The main effort of BioCreative experiments is to provide as much as possible realistic biological scenarios for performance assessment of automated annotation tools. The two GO annotation-specific subtasks at the most recent BioCreative IV [14] were aimed at assessing automated GO recognition, given as input *full-text articles* with relevant *gene information*: i) Task A. Retrieve GO evidence text for relevant genes (text retrieval task); ii) Task B. Predict GO terms for relevant genes (concept-recognition task). The performances were assessed with both gold-standard GO annotations and the help of expert GO curators. The overall conclusions of BioCreative’s assessors are that, despite the improvement over the last decade, even the best performing methods are still not accurate enough to aid manual GO curation.

In this work we focus on *GO annotation of biomedical literature*, namely the automatic assignment of GO terms to a scientific publication. This problem is closely related to BioCreative’s Task B, which further requires to identify the associations between GO annotations related to a publication and genes within its text. GO annotation of biomedical literature is itself a relevant sub-problem of the most general gene annotation task. First of all, divide-and-conquer strategies often reduce complexity for difficult problems. Therefore, decoupling the GO annotation task (for publications) from the GO association task (for genes within the publication) leads to two simpler subproblems, which could be approached with ad hoc techniques. Also, the unsatisfactory results obtained so far in automated annotation could be due to a lack of easily accessible gold-standard training data, such as full-text articles and evidence sentences related to GO terms and gene names. Conversely, public web repositories contain a growing number of heterogeneous metadata and annotations, which can be automatically processed by text annotation tools. Furthermore, the literature annotation problem is of interest in itself for the development of ontology based search engines, such as GoPubMed [15], which could be used as a pre-filter by human curators.

In the Information Retrieval (IR) community, GO annotation of biomedical literature can be seen as a *hierarchical multi-label classification* problem, where GO terms represent (a large number of) categories in which biomedical publications have to be classified [16]. There are two major approaches for hierarchical multi-label classification. In the *big-bang* approach a single supervised classification model is trained and used on the whole hierarchy [17–19]. The training of these methods becomes impractical at increasing number of categories. In the most common *top-down* approach a different classifier is used for each node of the hierarchy [20–22]. In these methods, an iterative process is carried on from the root of the hierarchy, progressively descending to more specific categories in order to detect the potentially correct one. Typical issues in the top-down approach involve the identification of the most promising paths to follow at higher levels of the hierarchy, as well as the detection of the levels at which to stop searching. More generally, classical IR approaches are *topic-centric*, in that they rely on indexed representations of the categories. Indexing is typically performed by using a controlled vocabulary of terms contained in pre-labeled documents. The classification accuracy drops down as the categories are poorly represented, thus state-of-the-art IR methods are best suited on hierarchies consisting of relatively few and well-represented categories. Some attempts have been made to apply supervised text classification methods in large taxonomies [21, 23, 24], but the results are still quite poor in comparison to those obtained in smaller settings. To our knowledge, the only available tool that directly addresses the hierarchical multi-label classification over GO categories is GOCat [25]. Differently from classical IR approaches, GOCat is *document-centric*, in that it uses a k-Nearest Neighbors (k-NN) strategy [26]. While in topic-centric approaches a query document is classified by means of direct comparisons with categories, with GOCat, annotations for a query abstract are inferred from the *k* most similar pre-annotated publications in a knowledge base. The k-NN approach proved to be valuable and best performing in one of the GO annotation subtasks (Task B) at BioCreative IV [27].

Here we introduce several new ideas for GO annotation of biomedical literature. First, we exploit a novel approach that combines the document-centric and topic-centric strategies. Second, we experiment with novel measures for assessing the similarity between documents and against category membership. Our resulting annotation tool, GOTA, makes use of publication title, abstract, references and year of publication (all readily available in public repositories, such as PubMed), although the approach itself is easily scalable to incorporate more (or less) information. We test the classification capabilities on a quite large set of scientific publications (15.000 documents) and with respect to different evaluation metrics. By comparison with GOCat, our approach shows better performance over all considered metrics. As a general consideration, the experimental results are encouraging and, in some aspects, surprising. In summary, on the average almost half of the gold standard annotations can be recovered for a query publication. This is a quite satisfactory result in comparison to other general-purpose hierarchical multi-label classification tools. More specifically, the annotations recovered for a publication, although not precise, are usually semantically close to the gold-standard annotations. Furthermore, we found that the classification capabilities improve over specie-specific knowledge bases. This suggests that the GO curation task could benefit from developing species-centered annotation tools. To our opinion, the most interesting findings are related to the smallest source of information that can aid classification. It is not unexpected that the biological content of a paper is summarized in few short sentences within the text, while the rest is background noise. In our experiments, given the information available for classification, it comes out that the strongest signal comes from the publication title.

## Methods

### Benchmark set

The Gene Ontology vocabulary was retrieved from Gene Ontology Consortium web resource [28]. At the time of the retrieval, the GO vocabulary consisted of 39,399 distinct terms partitioned into three main categories, structured as directed acyclic graphs (DAG) with a unique root: 26,099 terms of type Biological Process (BP), 9753 of type Molecular Function (MF) and 3547 of type Cellular Component (CC). The literature-based GO annotations were retrieved from the UniProt-GOA web resource [29]. We downloaded all the single Gene Association files, corresponding to set of proteins in different species/knowledge bases (30 overlapping sets). The single files were parsed in order to extract only the associations between pairs of GO terms and PubMed identifiers (PMIDs), discarding the gene product references. We ended up with a benchmark set of 328,357 pairs, consisting of 115,402 distinct PMIDs annotated with 22,839 distinct GO terms (see Additional file 1). The title, abstract and reference information related to the PMIDs in the benchmark set were downloaded in XML format from PubMed [30]. For 1256 out of 115,402 documents (1 %) the information downloaded from PubMed consists of the publication title only. For a total of 45,341 documents (39 %) also the cited references are available. Out of 22,839 GO identifiers in benchmark set, 14,889 are of type BP, 5951 of type MF and 1999 of type CC. The number of GO annotations for each PMID ranges from 1 to 1309 terms, with an average of 2.8. The distribution of annotations in the benchmark set is not uniform. In particular, except for a small subset, the number of annotations per PMID is quite small: 39 % of PMIDs have a single GO annotation and 99 % at most 10 distinct annotations (see Additional file 2).

Our method requires a preprocessing of the underlying Knowledge Base (KB) and a parameter tuning phase (see “Text preprocessing” section and “Tuning of parameters” section below). Both these phases are extremely time-consuming, which prevents the possibility of performing multiple rounds of cross validation in reasonable time. For this reason, for performance evaluation, we randomly selected a large sample of 15,000 publications from the benchmark set derived from UniProt-GOA. Publications in this set are used for testing the classification performances, while the remaining ∼100,000 publications constitute the underlying KB of our method. Almost 6 % of the terms represented in the test set have no associated publication in the KB.

Among these *missing* terms, there is a higher number of BP terms (67 %) in comparison to MF (25 %) and CC (8 %) terms.

### Approach

We use the combination of a publication-centric and term-centric approach to capture the relation between a scientific publication and a GO term:

1. *Publication-centric*: the relation between a GO term *t* and a query *q* is inferred from the query’s similarity with annotated publications in the underlying KB.
2. *Term-centric*: the relation between the topics covered by a query *q* and a GO term *t* is determined by direct comparison.

**Fig. 1:**
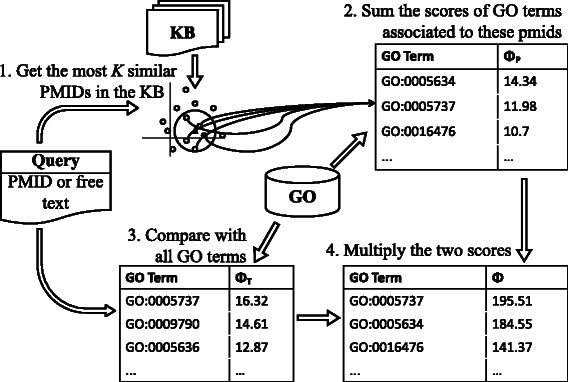
GOTA workflow Graphical representation of Eq. 1

The likelihoods obtained from these two approaches are simply combined into a single relatedness score:

(1) $$ \Phi(q,t) = \Phi_{P}(q,t) \cdot \Phi_{T}(q,t),  $$

where *Φ*_*P*_ and *Φ*_*T*_ are the *publication-centric* and the *term-centric* similarity scores, respectively. The meaning of Eq. 1 is quite simple (see Fig. 1). The *Φ*_*P*_ function makes use of a k-NN strategy to select a ranked list of GO terms (only terms associated to some publication in the KB can be selected). The *Φ*_*T*_ function provides a re-weighting of the selected terms.

The details of the preprocessing and tuning phase, as well as the exact definition of the two similarity functions in Eq. 1 are given in the rest of this section.

#### Text preprocessing

We perform typical preprocessing operations to transform each publication and GO term into a structured representation more manageable for querying. We make use of the quite common *Bag of Words* (BoW) representation of documents. The BoW representation describes a textual document by means of a feature vector, where each entry indicates the presence or absence of a word. The elements of the vector are weighted in order to balance their relative importance. Other than BoW representation, we experimented with different features that can be automatically extracted from PubMed and GO knowledge bases. In detail, a publication *p* is indexed by the following information:

- $\mathcal {W}(p)$ (*text*): BoW built from the abstract and title of the publication *p*. More generally, this feature represents the BoW built from unstructured text associated to the publication.
- $\mathcal {T}(p)$ (*title*): BoW built from the title of the publication *p*.
- $\mathcal {R}(p)$ (*references*): weighted vector of references (PMIDs). As for the BoW, each entry of the vector indicates the presence (or absence) of a reference within the publication. The references are weighted according to their importance.
- $\mathcal {Y}(p)$ (*year*): year of publication of *p*.

All the features above can be easily extracted for documents indexed into PubMed. Some of these features could not be available for some publication (references, for example), particularly for query publications. We assume that at least the $\mathcal {W}(p)$ feature vector is always non-null (i.e. contains entries greater than zero). A GO term *t* is indexed with the following information (with abuse of notation, we use the same symbol when the term-specific and publication-specific features are related):

- $\mathcal {W}(t)$ (*text*): BoW built from unstructured text associated to the term *t*. In this case, the text associated to *t* includes the term name, synonyms and description (all available in the GO knowledge base), as well as all the titles and abstracts of those publications in the KB associated to term *t* (these informations could be absent).
- $\mathcal {T}(t)$ (*name*): BoW built from the name and synonyms of the term *t*.
- $\mathcal {Y}(t)$ (*year*): average year of the publications annotated with term *t*.

The $\mathcal {W}(t)$ and $\mathcal {T}(t)$ BoWs are always non empty, while the $\mathcal {Y}(t)$ feature can be unspecified (if there is no publication in the KB annotated with *t*).

We use the following procedure to create a pool of words (BoW) appearing in titles and abstracts of publications in the KB, as well as title, synonyms and descriptions of GO terms. Single words occurring in the text are extracted, discarding punctuation. A stopword list is used to filter-out all the words that are not enough informative about the text (such as, *actually, after,* etc). The standard Porter stemming algorithm [31] is used to group words with common stems. As the method works accurately even without filtering or selecting features, which generally are costly steps that require further complex tuning of parameters, we do not perform such task in this paper. However, we use the common *tf-idf* scheme [32], which is one of the several existing techniques [33], to assign a relevance weight to words related to each feature.

The *Term Frequency* (*tf*) measures how frequently a word appears into a document:

$$tf(w,x) = \frac{\text{number of times word}\ w\ \text{appears in text}\ x}{\text{total number of words in text}\ x} $$

The *Inverse Document Frequency* (*idf*) measures how much important a word is:

$$ {\small{\begin{aligned} &idf(w) \\ &= \log\!{\frac{\text{total number of publications and GO terms}}{\text{number of publications and GO terms containing}\ w}} \end{aligned}}}  $$

The *tf-idf* scheme is the product of these two quantities. To automatically extract weighted vectors of references $\mathcal {R}(p)$, we use the same criteria of the *idf* measure. Publications indexed in PubMed are identified with a unique PMID and cited references are represented as a list of PMIDs. Thus, the *idf* measure for references can be easily calculated by:

$$ {\small{\begin{aligned} &{idf}_{\text{ref}}({\text PMID})\\ &\quad=\log\frac{\text{total number of publications with { bibliography}}}{\text{number of publications citing PMID}} \end{aligned}}}  $$

With the BoW representation, the similarity between two feature vectors *x*,*y* can be measured by means of the commonly used *cosine similarity* [34]:

$$sim_{cos}(x,y)=\frac{x\cdot y}{\| x\| \|y\|} $$

Since by definition the BoW vectors have only positive values, the *s* *i* *m*_*cos*_ score is always non-negative.

#### Publication-centric score

The first similarity score between a publication query and a GO term is recovered by comparison with annotated literature, namely the publications in the underlying KB. This is basically a k-NN approach. First, the publication query is compared with all the publications in the KB, in order to detect those that appear to be most closely related to the query (Step 1 in Fig. 1). Second, a ranked list of GO annotations for the query is inferred from the recovered publications (Step 2 in Fig. 1).

The comparison score between a query publication *q* and a target publication *p* in the KB is given by the product of four different similarity measures:

(2) $$  \phi_{P}(q,p)= \Pi_{i=1}^{4} (1+f_{i}(q,p))^{m_{i}}  $$

where 0≤*f*_*i*_(*q*,*p*)≤1 gives a similarity score associated to a single publication-related feature and the power *m*_*i*_ represents its relative weight. The four similarity functions *f*_*i*_ *q* *p* are defined by:

1. (*Text similarity*) Cosine similarity between BoW of the query and target publications:
  $$f_{1}(q,p)=sim_{cos}(\mathcal{W}(q),\mathcal{W}(p)) $$
2. (*Title similarity*) Cosine similarity between the title-related BoW of the query and target publications:
  $$f_{2}(q,p)=sim_{cos}(\mathcal{T}(q),\mathcal{T}(p)) $$
3. (*References similarity*) Cosine similarity between the weighted vector of references of the query and target publications:
  $$f_{3}(q,p)=sim_{cos}(\mathcal{R}(q),\mathcal{R}(p)) $$
4. (*Year similarity*) Distance between the publication year of the query and target. We decided to normalize this value with a maximum distance of 50 years:
  $${}f_{4}(q,p)\! =\!\! \left\{\! \begin{array}{l l} 0 & \text{if}\ |\mathcal{Y}(q)-\mathcal{Y}(p)|>50\\ (50\,-\,|\mathcal{Y}(q)-\mathcal{Y}(p)|)/50 & \text{otherwise} \end{array} \right.$$

We remark that the four features described above can be used for the classification only if the query publication is indexed into PubMed. In all the other cases, i.e. when the query consists of unstructured text only, the only available feature is *f*_1_(*q*,*p*). Furthermore, also for publications indexed in PubMed some information can be missing (abstract and/or bibliography, for example). However, due to the definition of Eq. 2, the missing information does not affect the calculation of the comparison score. Symmetrically, the comparison score can be easily extended to incorporate more features.

The measure *ϕ*_*P*_(*q*,*p*), computed by Eq. 2, is used to rank the publications in the KB according to their similarity with the query. The gold-standard GO annotations of the top-*K* ranked publications are then transferred to the query, by using *ϕ*_*P*_(*q*,*p*) as their relative weight. The *publication-centric similarity score* between a query *q* and a GO term *t* is then given by:

(3) $$ {}\Phi_{P}(q,t) \,=\, \sum_{\text{top}-\textit{K} \text{ranked}\ p \in KB} \left\{\! \begin{array}{ll} \phi_{P}(q,p) & \text{if}\ p\ \text{has annotation}\ t \\ 0 & \text{otherwise} \end{array} \right.  $$

#### Term-centric score

The second similarity score between a publication query and a GO term is based on the direct comparison between a query *q* and a term *t* (Step 3 in Fig. 1). Also in this case, the score is given by the the product of different similarity measures:

(4) $$ \Phi_{T}(q,t)= \Pi_{i=1}^{5} (1+g_{i}(q,t))^{n_{i}}  $$

where 0≤*g*_*i*_(*q*,*t*)≤1 is the score associated to a single GO term-related feature and the power *n*_*i*_ represents its relative weight. The five similarity functions *g*_*i*_(*q*,*t*) are defined by:

1. (*Text similarity*) Cosine similarity between the BoW of the query and term:
  $$g_{1}(q,t) = sim_{cos}(\mathcal{W}(q),\mathcal{W}(t)) $$
2. (*Title similarity*) Cosine similarity between the title-related BoW of the query and term:
  $$g_{2}(q,t)=sim_{cos}(\mathcal{T}(q),\mathcal{T}(t)) $$
3. (*Name frequency*) Percentage of the words in the title-related BoW of the term that are contained in the BoW of the query:
  $$g_{3}(q,t) = \frac{|\mathcal{T}(t) \cap \mathcal{W}(q)|}{|\mathcal{T}(t)|} $$
4. (*Name occurrence*) Normalized number of occurrences of the name or synonym of the term in the query. In this case, we seek the full name of the term (and its synonyms) in the entire query text. The number of occurrences *c* is normalized to 4.
  $$g_{4}(q,t) = \left\{ \begin{array}{ll} 1 & \text{if}\ c>4\\ c/4 & \text{otherwise} \end{array} \right. $$
5. (*Year similarity*) Distance between the publication year of the query and term:
  $${}g_{5}(q,t)\! = \!\left\{\! \begin{array}{l l} 0 & \text{if}\ |\mathcal{Y}(q)-\mathcal{Y}(t)|\!>50\\ (50\,-\,|\mathcal{Y}(q)-\mathcal{Y}(t)|)/50 & \text{otherwise} \end{array} \right. $$

### Tuning of parameters

The computation of Eq. 1 requires a preprocessing of the underlying KB, as well as the tuning of a few parameters. The parameter tuning has been performed over the 100,402 publications in the KB, which has been randomly extracted from the 115,402 publications of the benchmark text set. Moreover we randomly split the KB into two text sets: a training set of 90,402 publications and a validation set of 10,000 publications.

Generally the tuning of parameters requires performing a cross-validation strategy, however, as previously explained, we do not use such a strategy because of (i) the computational time required for preprocessing a so large data set and because (ii) the amount of data, randomly selected for the validation set, is representative of the entire benchmark text set. In fact, according to the sampling theory [35], in the worst case the representativeness (or sampling) error of this validation set is less than 0.98 %, with a confidence level of 95 %. We also remark that the effectiveness of the method does not strongly depend from the parameter tuning. In fact, without it the drop in classification accuracy over the validation set is less than 2 % (data not shown).

The parameters involved in the tuning process belong to the two functions that calculate the two similarity scores. In particular the computation of the similarity score *Φ*_*P*_(*q*,*t*) in Eq. 3 requires the tuning of the power parameters *m*_*i*_ in Eq. 2 and the *K* parameter in Eq. 3. We tested all the combinations of the *m*_*i*_ ranging from 0 to 4 each. Note that a 0-value leads to not considering the related feature.

The selected power parameters are: *m*_1_ = 4,*m*_3_ = 3,*m*_2_ =*m*_4_ =1. While the power parameters seem to give more importance to some similarity scores, such as *f*_1_(*q*,*p*), they actually play a balancing role, since the actual values of the four similarity scores follow different distributions. We also remark that, when the input query consists uniquely of unstructured text, the classification relies only on the *f*_1_(*q*,*p*) similarity score. In such situations, the power parameter *m*_1_ has only a limited effect on the scoring. We also tested the method by varying the number (*K*) of most similar publications in the KB considered in Eq. 3. We experimented that varying *K* from 50 to 1000, we obtain the best results with *K* ranging from 50 to 300, with a maximum peak at *K*=150.

As for Eq. 3, we tuned the power parameters *n*_*i*_ for the Eq. 4 testing all combinations with each value ranging from 0 to 4. The selected parameters are: *n*_1_=4,*n*_3_=2, *n*_2_ = *n*_4_ = *n*_5_ = 1. When the input query consists just of unstructured text, the only power parameters used are *n*_1_,*n*_3_ and *n*_4_.

We further considered the problem of assigning a confidence threshold (low, medium, high) to the predicted annotations. The aim of such filter is to provide to the user a confidence level, mainly for those queries that have a very low biological content. The confidence score is not considered for the experimental testing in the paper, but it is made available on the online Web application. Further details are reported in Additional file 2.

### Evaluation metrics

In a real world scenario, a text mining tool for GO classification should assist a database curator for manual extraction of GO annotations from scientific literature. From this point of view, a classification tool can be useful whether it can provide a small set of annotations that accurately cover the biological content of a scientific publication. Thus, for performance assessment, we particularly focus on the evaluation of the top-10 ranked terms only (recall that 99 % of the publications in our benchmark set have at most 10 distinct GO annotations). The performances for the top-20 ranked terms are shown in Additional file 2.

Furthermore, in order to make the results more accessible to a heterogeneous community, we adopt different metrics from different domains. In particular, we use specific evaluation metrics from the IR domain, as established at TREC experiments [36], and metrics exploited for performance assessment in biologically-related domains, such as BioCreative [14] and CAFA [5] experiments. The topic covered at CAFA is the evaluation of protein function prediction methods. As in our setting, also at CAFA the performances are assessed over the GO hierarchy. We further introduce a third set of metrics, based on recent approaches for measuring semantic similarity between set of terms/concepts [37].

In the rest of this section, we adopt the following notation. For a given query, we denote with *T* and *P* its related set of gold-standard (from GOA) and predicted (from the classifier) GO annotations, respectively. We use lowercase letters, such as *t* and *p*, to denote single terms within *T* and *P*, respectively. We can assume that predicted terms in *P* are ranked according to the classifier score. We denote with *P*_*k*_ the top-*k* ranked terms in *P*. With abuse of notation, we use *P*_*s*_ to denote also the subset of predicted terms in *P* with a score greater than or equal to score threshold *s*. A detailed description of the metrics follows.

#### TREC metrics

The *reciprocal rank* metric evaluates the precision at first correctly predicted term by computing the reciprocal of its rank:

(5) $$ {RR}_{k}(T,P)= \frac{1}{\min\{ i \mid T\cap P_{i}\neq \emptyset, 1\leq i\leq k\}}.  $$

The *M* *R* *R*_*k*_ is computed by averaging the *R* *R*_*k*_ over the entire set of evaluated queries. The *recall at rank k* metric evaluates the average fraction of relevant GO terms in the top-*k* predictions returned by the classifier:

(6) $$ R_{k}(T,P) = \frac{|T\cap P_{k}|}{|T|}.  $$

The *R* *R*_*k*_ and *R*_*k*_ measures are strictly greater than zero only if *T*∩*P*_*k*_≠*∅*. For performance evaluation, here we consider only the mean reciprocal rank and recall at the top-10 predictions, *M* *R* *R*_10_ and *R*_10_, respectively.

#### CAFA/BioCreative metrics

These metrics, introduced to reflect the hierarchical nature of GO, are based on a variant of the standard precision/recall measures, called *hierarchical precision/recall* [38]. The hierarchical measures are some of the metrics adopted at the BioCreative IV experiments. The hierarchical-precision at rank *k* is defined by

(7) $$ {hP}_{k}(T,P) = \frac{|\mathcal{A}(T)\cap \mathcal{A}(P_{k})|}{|\mathcal{A}(P_{k})|},  $$

and the hierarchical-recall at rank *k* by

(8) $$ {hR}_{k}(T,P) = \frac{|\mathcal{A}(T)\cap \mathcal{A}(P_{k})|}{|\mathcal{A}(T)|},  $$

where $\mathcal {A}(X)$ denotes the set of all the ancestors of terms in *X*, recursively propagated up to the root(s) of the GO hierarchy. The set $\mathcal {A}(X)$ contains also *X*, i.e. $X\subseteq \mathcal {A}(X)$. The hierarchical-precision and hierarchical-recall can be combined into a single metric, the hierarchical *F*-measure (harmonic mean):

(9) $$ {hF}_{k}(T,P) =\frac{2\cdot {hP}_{k}(T,P) \cdot {hR}_{k}(T,P)}{hP_{k}(T,P) +{hP}_{k}(T,P)}  $$

The *h* *P*_*k*_(*T*,*P*) measure tends to assign high scores when *P* contains very generic terms, such as those at the top of the hierarchy. Thus, the *h* *P*_*k*_(*T*,*P*) measure is not very robust over GO, since it gives high scores even when *P* consists of just few non-informative terms, such as the three roots of the GO hierarchy. Symmetrically, the *h* *R*_*k*_(*T*,*P*) measure tends to assign high scores when *P* contains very specific terms, such as those at the bottom of the hierarchy. Since the GO hierarchy contains many leaf terms, the *h* *R*_*k*_ measure is more robust than the *h* *P*_*k*_ over GO. Furthermore, if we choose a fixed *k* (= 10 or 20), even if *T*⊂*P*, *h* *P*_*k*_(*T*,*P*) would generally provide a poor estimation of the classification capabilities, due to the highly unbalanced number of annotations per publication in our benchmark set. Conversely, the unbalanced number of annotations does not affect the *h* *R*_*k*_ metric. The *h* *F*_*k*_ metric is more robust than *h* *P*_*k*_ but it still suffers from the unbalanced amount of annotations in our dataset. For these reasons, here we consider only the hierarchical recall at rank 10, *h* *R*_10_. The results for *h* *P*_10_ and *h* *F*_10_ are shown in Additional file 2.

With small modifications, the hierarchical measures have been adopted also at CAFA experiments. In detail, at CAFA, the top-ranked predictions are selected with respect to a given score threshold *s*, not a rank *k*. The hierarchical precision and recall in Eqs. 7 and 8, respectively, just need to be recoded with respect to score thresholds. Note that, in $ \mathcal {A}(P_{s})$ only the predicted terms with score greater than or equal to *s* are propagate up to the root(s) of the ontology. Furthermore, the hierarchical precision *h* *P*_*s*_(*T*,*P*) is assumed to be equal to zero if |*P*_*s*_|=0. For a given dataset *D* consisting of pairs of true and predicted annotations, and for a given score threshold *s*, CAFA’s average hierarchical precision at *s* is defined by

$${hP}_{s}(D) = \frac{1}{m_{s}(D)}\sum_{(T,P)\in D} {hP}_{s}(T,P), $$

where *m*_*s*_(*D*)=|{*P*|(*T*,*P*)∈*D*,|*P*_*s*_|>0}| is the number of non-empty predictions at threshold *s*. Asymmetrically, the average hierarchical recall at threshold *s* is averaged over all pairs in the dataset, and it is defined by

$${hR}_{s}(D) = \frac{1}{|D|}\sum_{(T,P)\in D} {hR}_{s}(T,P). $$

These last two measure can be combined into a single metric, the *F*-measure (harmonic mean), at different score thresholds. The main evaluation metric at CAFA, which we also consider here, is the maximum value of the harmonic mean over all thresholds:

(10) $$ {hF}_{max}(D) = \max_{s} \left\{ \frac{2\times {hP}_{s}(D) \times {hR}_{s}(D)}{hP_{s}(D) + {hR}_{s}(D)} \right\}.  $$

Thanks to the choice to adopt a score cutoff, CAFA’s hierarchical *F*-measure is not affected by the unbalanced number of annotations in the benchmark set.

#### Information-Theoretic metrics

These metrics can be considered the information-theoretic counterparts of precision/recall, and rely on the information content of individual terms *t* in the GO hierarchy:

$$ic(t)=-\log Pr(t) $$

where *P* *r*(*t*) is the relative frequency of term *t* with respect to some background distribution. We adopt as background distribution the entire set of gold-standard annotations in our benchmark set. The Resnik’s similarity [39] between two terms *t* and *p* is defined as the maximum information content among the common ancestors of *t* and *p*:

$${sim}_{Resnick}(t,p)=\max_{a \in \mathcal{A}(\{t\}) \cap \mathcal{A}(\{p\})}\{ic(a)\} $$

The Lin’s similarity [40] between two terms *t* and *p* is the normalized version of the Resnick’s similarity:

$${sim}_{Lin}(t,p)=\frac{2\times {sim}_{Resnick}(t,p)}{ic(t)+ic(p)} $$

Following the approach in [37], we can extend the information-theoretic similarities to sets of annotations. In this way, it is possible to obtain the information-theoretic counterpart of precision at the top-*k* ranked terms:

(11) $$ {iP}_{k}(T,P)=\frac{1}{|\mathcal{L}(P_{k})|}\sum_{p\in \mathcal{L}(P_{k})} \max_{t\in \mathcal{L}(T)}\{sim_{Lin}(t,p)\},  $$

and recall at the top-*k* ranked terms:

(12) $$ {iR}_{k}(T,P)=\frac{1}{|\mathcal{L}(T)|}\sum_{t\in \mathcal{L}(T)} \max_{p\in \mathcal{L}(P_{k})}\{sim_{Lin}(t,p)\}  $$

where $\mathcal {L}(X)$ denotes the set of *leaf terms* in *X*, i,e. $\mathcal {L}(X)\subseteq X$ is the largest subset of *X* such that $\forall u,v \in \mathcal {L}(X), u\notin \mathcal {A}(v)$. As it happens for the hierarchical-precision *h* *P*_10_, the information-theoretic precision *i* *P*_10_ is slightly affected by the unbalanced number of annotations in our benchmark set. Conversely, the *i* *P*_1_ metric is more robust, since it just estimates the quality of the very top predicted annotation. For these motivations, here we consider only the information-theoretic precision at the top ranked term (*i* *P*_1_) and the information-theoretic recall at rank 10 (*i* *R*_10_), which can be seen as complementary to the two TREC metrics *M* *R* *R*_10_ and *R*_10_. The results for *i* *P*_10_ are shown in Additional file 2.

## Results and discussion

In the following sections, performances are assessed over the entire GO hierarchy, without considering separately the three main ontologies BP, MF and CC. The detailed results can be found in Additional file 2. As a general consideration, the classification accuracy is overall better if we asses the results separately for the three ontologies. Furthermore, it is higher over the MF and CC in comparison to BP. These results are not completely unexpected, since the entire GO hierarchy contains much more categories/terms than its main sub-ontologies. Equivalently, the number of distinct categories is much lower in MF and CC than in BP. Furthermore, also baseline random classifiers have surprisingly good classification performances over MF and CC (see Additional file 2). This may suggest that there is some manual-annotation bias toward few specific MF and CC terms. Conversely, the performance gap between random and non-random classifiers is much more pronounced over the entire GO hierarchy. The results over the complete GO are thus more representative of the true classification limits of the different approaches.

### Overall classification performances

Classification performances on our test set of 15,000 publications (see “Benchmark set” section) are shown in the top section of Table 1. In order to clarify how the classification is affected by different sources of information, in Table 1 we show the performances of our best-parameter model, which makes use of the detailed information available in PubMed (PM), and those of two more versions that make use of unstructured text only: title plus abstract (T + A), and only title (T). In these last two models, the classifier is queried with unstructured text and it does not know whether the input text is related to the publication title and/or abstract. We include in the comparison also two naive baseline predictors obtained from the distribution of GO terms in our KB. Both naive predictors assign exactly the same predictions to all query targets. The first naive predictor, RandFR, ranks the GO terms according to their relative frequency in the underlying KB. The second naive predictor, RandIC, ranks each GO term according to its average information-theoretic precision (Eq. 11), with respect to all the publications in the underlying KB.

**Table 1:** Performances over a test set of 15,000 publications

Method ^*a*^	Info ^*b*^	IT ^*c*^		CAFA ^*c*^	BC ^*c*^	TREC ^*c*^	
		*i* *P*_1_	*i* *R*_10_	*h* *F*_*max*_	*h* *R*_10_	*M* *R* *R*_10_	*R*_10_
GOTA	PM	*0.43*	*0.64*	*0.43*	*0.69*	*0.40*	*0.46*
GOTA	T+A	0.42	*0.64*	*0.43*	0.68	0.39	0.45
GOTA	T	0.41	0.63	0.42	0.68	0.39	0.44
RandFR	N/A	0.20	0.33	0.20	0.33	0.18	0.15
RandIC	N/A	0.21	0.27	0.18	0.31	0.03	0.08
GOTA *Φ*_*P*_	PM	0.37	0.64	0.41	0.67	0.38	0.44
GOTA *Φ*_*P*_	T+A	0.35	0.62	0.40	0.66	0.36	0.41
GOTA *Φ*_*P*_	T	0.35	0.62	0.40	0.66	0.36	0.41
GOTA *Φ*_*T*_	PM	0.28	0.41	0.30	0.49	0.16	0.17
GOTA *Φ*_*T*_	T+A	0.24	0.37	0.27	0.46	0.11	0.12
GOTA *Φ*_*T*_	T	0.22	0.35	0.26	0.44	0.09	0.10

^*a*^Method used for the classification. RandFR and RandIC are baseline predictors, based on the distribution of GO terms in the training set

^*b*^Informations used in prediction: PM = title, abstract, references and publication year (PubMed); T+A = title and abstract; T = title; N/A = no information

^*c*^Metrics definitions are in the “Evaluation metrics” section. In top section of the table, for each metric, the best result is highlighted in italic

As shown in Table 1, GOTA classification capabilities are much better than those of the two naive predictors, over all considered metrics. By observing in more detail the best performances (first row) in Table 1, according to the *M* *R* *R*_10_ metric, on the average a prediction includes a gold standard annotation at rank between 2 and 3. According to the *R*_10_ metric, 46 *%* of the gold standard annotations are included in the top-10 predicted terms. Although not directly comparable, these results are better that those reported at BioCreative IV Task B [14] and for other large hierarchical multi-label classification settings [24]. The information-theoretic measures provide more interesting insights into the classification capabilities. According to the *i* *R*_10_ metric, the average semantic similarity between the gold standard annotations with respect to the top-10 predicted terms is 0.64. According to the *i* *P*_1_ measure, the average semantic similarity of the top predicted term is 0.46. By themselves these scores say little about the quality of the predicted annotations. However, we can easily calculate some statistics to asses whether these values are meaningful or not. For 72 % of the gold-standard terms (represented in the test set) there are less than 1 % terms (represented in the KB) with semantic similarity ≥0.46. That is, two terms with semantic similarity ≥0.46 are very likely to be closely related. We also bootstrapped (10,000 draws of 10 terms each from the KB) the sample mean and standard deviation of the *i* *R*_10_ and *i* *P*_1_ scores for each test-publication. At a confidence level of 10^−5^, GOTA’s *i* *R*_10_ and *i* *P*_1_ scores are significantly higher (according to a Z-test) than the samples means for 88 and 57 % of the test publications, respectively. The conclusion is that, on the average: i) the top-10 predicted terms include GO terms that are significantly similar to the gold standard annotations. ii) such classifications are very unlikely to be observed by random selection of GO terms from the KB. With respect to the *h* *R*_10_ metric, we can observe a satisfactory performance (although not directly comparable) with respect to the results reported at BioCreative IV Task B [14]. Finally, although protein and literature annotation are different but related problems, the *h* *F*_*max*_ measurements for BP and MF (see Additional file 2) are higher in comparison to the best performing methods at CAFA [5]. With respect to CAFA, we can report better results also if we restrict to subsets related to specific species (see Human, Mouse and Rat performances in Additional file 2 and Supplementary Information of [5]). This may suggest that the automated GO annotation task from protein sequences is an even harder problem than literature-based annotation.

Although the classifier that makes use of more detailed information (PM) has the best performances, its classification capabilities are not much higher than those of two models that make use of unstructured text only (T + A and T). We can asses whether the improvement is statistically significant with a paired Student’s *t*-test over all selected metrics but *h* *F*_*max*_ (which is not an averaged score). At standard significance level of 5 % (the detailed *p*-values are shown in Additional file 2), the performances of the full model (PM) are significantly better over all metrics in comparison to the title plus abstract-based model (T + A) and the title-based model (T). However, the performances of the two text-based classifiers are indistinguishable for some of the adopted metrics (data not shown). This is somewhat surprising and suggests that publication title is the most important source of information for GO annotation of scientific literature.

Another interesting question is whether authorship can introduce some bias in the experimental testing. In particular, we ask to which extent having in test and training papers from the same author(s) can affect the classification performance. In this context, the authorship information can be relevant for two main reasons: i) the way in which an author writes can be repetitive in some parts and it could affect the text similarities extracted by an automatic classifier, and ii) it is conceivable that the same authors could work and publish more than one papers on similar topics. In Additional file 2 we show some experiments that exploit the authorship information. The overall conclusion of these tests is that authorship information alone provides better results than random classification. On the other end, such information does not affect significantly GOTA’s performances.

To conclude, in the bottom section of Table 1 we show the average performances of the two comparison scores (*Φ*_*P*_ and *Φ*_*T*_) used in the GOTA classifier (see “Approach” section). It is evident that publication-centric approach (*Φ*_*P*_) is more accurate than the topic-centric approach (*Φ*_*T*_). However, their combination provides overall better results. This shows that the two approaches are not significantly affected by collinearity problems. In fact, their two BoWs are built using different techniques.

### Performances on species-specific knowledge bases

A natural question concerning GO annotation of literature is whether we can improve the classification accuracy by restricting to species-specific knowledge bases. For instance, if we know a-priori that some publication is related to some specific species, for annotation purposes is it better to compare it with a smaller set of publications on the same species or with a larger set of publications on different species?

In order to answer this question, here we considered four species for which we found a sufficiently high number of annotated publications in GOA database: Human (27,133 distinct publications, 24 *%* of the entire benchmark set), Mouse (21,581, 19 *%*), Rat (18,321, 16 *%*) and Yeast (10,162, 9 *%*). We extracted from our test set of 15,000 publications the subsets related to the selected species. For each one of such subsets we compare the performances we obtain when the underlying KB is the same as in previous Section (Full) and when it consists of species-specific subsets only (Human, Mouse, Rat and Yeast). The species-specific subsets have been constructed by selecting from the KB only the PMID-GO associations listed in the species-related GOA files. The results are summarized in Table 2, where we can observe that the classification performances over species-specific KBs are overall better than those on the full KB. However, the improvement is statistically significant (as assessed by a paired *t*-test at significance level of 5 %) over almost all the adopted metrics only on the set of publications related to Rat and Yeast. In the remaining cases, the improvement is not uniformly significant for all the considered metrics, but only some of them (the detailed *p*-values are shown in Additional file 2). These results indicate that, in some specific cases, specie-related KB can help to improve dramatically the classification accuracy. To some extend, these results are not completely unexpected, since specie-specific KBs identify specie-specific subsets of GO terms. For instance, the rat-specific KB collects 9090 distinct terms, in comparison to the 25,622 terms in the full KB. Thus, the classification of rat-related publications is less challenging, since the rat-specific KB drastically reduces the number of possible categories represented in the full KB.

**Table 2:** Performance comparison over species-specific knowledge bases

Species ^*a*^	KB ^*b*^	Info ^*c*^	IT ^*d*^		CAFA ^*d*^	BC ^*d*^	TREC ^*d*^	
			*i* *P*_1_	*i* *R*_10_	*h* *F*_*max*_	*h* *R*_10_	*M* *R* *R*_10_	*R*_10_
Human	Human	PM	*0.45*	*0.62*	*0.46*	*0.69*	*0.49*	*0.49*
Human	Full	PM	0.44	*0.62*	0.44	*0.69*	0.44	0.48
Human	Human	T	0.42	0.60	0.45	0.66	0.46	0.47
Human	Full	T	0.44	0.61	0.44	0.68	0.45	0.47
Mouse	Mouse	PM	*0.45*	*0.63*	*0.45*	*0.67*	*0.45*	*0.44*
Mouse	Full	PM	*0.45*	0.61	0.44	0.66	0.43	0.42
Mouse	Mouse	T	0.42	0.63	0.44	0.65	0.43	0.42
Mouse	Full	T	0.44	0.60	0.43	0.64	0.42	0.41
Rat	Rat	PM	*0.38*	*0.64*	*0.41*	*0.69*	*0.36*	0.44
Rat	Full	PM	0.34	0.61	0.37	0.67	0.33	0.42
Rat	Rat	T	0.37	0.62	0.40	0.67	0.34	0.42
Rat	Full	T	0.33	0.61	0.37	0.66	0.33	0.42
Yeast	Yeast	PM	*0.45*	*0.72*	*0.47*	*0.77*	*0.42*	*0.50*
Yeast	Full	PM	0.43	0.70	*0.47*	0.75	0.39	0.49
Yeast	Yeast	T	0.41	0.68	0.44	0.74	0.37	0.45
Yeast	Full	T	0.41	0.68	0.44	0.73	0.35	0.46

^*a*^Only publications related to the specified Species are considered for the evaluation. Human: 3575 publications; Mouse: 2825 publications; Rat: 2380 publications; Yeast: 1290 publications

^*b*^Knowledge base used for prediction. Full = all available publications in the KB. Human/Mouse/Rat/Yeast = only publications related to Human/Mouse/Rat/Yeast

^*c*^Informations used in prediction: PM = title, abstract, references and publication year (PubMed); T = title

^*d*^Metrics definitions are in the “Evaluation metrics” section. For each metric and Species, the best result is highlighted in italic

As a final remark, as shown in Table 2, also for species-specific classification tasks, the publication title is the most important source of information. This is true irrespectively of the considered species.

### Performance comparison with related approaches

We compare the performances of our tool with GOCat [25] (ML classifier), which is, to date, the only publicly available tool closely related to our work. For comparison purposes, we extracted from our test set of 15,000 publications the set of 412 PMIDs not included in the KB of GOCat’s latest release. GOCat’s predictions over the 412 queries have been obtained by using the publication title only (T) and publication title plus abstract (T + A), in form of unstructured text. For a fair comparison, we removed from GOCat’s predictions all the terms not included in our GO hierarchy.

The results are summarized in Table 3. The performances of both GOTA and GOCat are significantly higher than those of the two naive classifiers RandFR and RandIC. GOTA performances with full information (PM) are significantly better then those of GOCat (T + A) over all the considered metrics, as assessed by a paired *t*-test at significance level of 5 % (the detailed *p*-values are shown in Additional file 2). GOTA makes use of more specific information than GOCat, such as references and publication year. Anyway, even when exactly the same information is used in prediction (T +A and T), GOTA classification capabilities are significantly superior to those of GOCat for almost all the considered metrics (data not shown). As a further analysis, we calculate what is the fraction of publications for which the top-10 predictions contain at least one gold standard term (see Additional file 2). We have an a amount of 62 *%* publications with GOTA against 52 *%* of GOCat.

**Table 3:** Performance comparison with different approaches

Method ^*a*^	Info ^*b*^	IT ^*c*^		CAFA ^*c*^	BC ^*c*^	TREC ^*c*^	
		*i* *P*_1_	*i* *R*_10_	*h* *F*_*max*_	*h* *R*_10_	*M* *R* *R*_10_	*R*_10_
GOTA	PM	*0.42*	*0.69*	*0.42*	*0.73*	*0.39*	*0.49*
GOTA	T+A	0.37	0.68	0.41	*0.73*	0.35	0.48
GOTA	T	0.39	0.66	0.39	0.70	0.34	0.44
GOCat	T+A	0.34	0.64	0.37	0.69	0.29	0.40
GOCat	T	0.30	0.64	0.36	0.69	0.28	0.40
RandFR	N/A	0.08	0.21	0.10	0.23	0.03	0.05
RandIC	N/A	0.22	0.23	0.19	0.30	0.00	0.01

^*a*^Method used for prediction. RandFR and RandIC are baseline predictors, based on the distribution of GO terms in the training set

^*b*^Informations used in prediction: PM = title, abstract, references and publication year (PubMed); T+A = title and abstract; T = title; N/A = no information

^*c*^Metrics definitions are in the “Evaluation metrics” section. For each metric, the best result is highlighted in italic

Analyzing in further detail GOTA and GOCat performances, we can verify whether they behave in similar or completely different way. In Table 4, for each evaluation metric (but *h* *F*_*max*_) we show the fraction of publications on which GOTA gets a score exactly equal to (GOTA = GOCat), strictly higher than (GOTA > GOCat) or strictly lower than (GOTA < GOCat) GOCat’s. Although GOTA’s performances are overall better, from Table 4 it is clear that there is a non-trivial fraction of publications on which GOCat performs better than GOTA.

**Table 4:** 1-to-1 comparison between GOTA (PM) and GOCat (T+A)

Metric ^*a*^	GOTA = GOCat ^*b*^	GOTA > GOCat ^*c*^	GOTA < GOCat ^*d*^
*i* *P*_1_	0.44 (0.13)	0.36 (0.15)	0.20 (0.07)
*i* *R*_10_	0.29 (0.16)	0.41 (0.15)	0.30 (0.07)
*h* *R*_10_	0.41 (0.25)	0.36 (0.17)	0.23 (0.09)
*M* *R* *R*_10_	0.42 (0.13)	0.37 (0.16)	0.20 (0.07)
*R*_10_	0.61 (0.21)	0.25 (0.17)	0.13 (0.08)

^*a*^Metrics definitions are in the “Evaluation metrics” section

^*b*^Fraction of publications on which GOTA and GOCat get exactly the same score. In parenthesis, fraction of publications on which the score is equal to 1 (maximum)

^*c*^Fraction of publications on which GOTA gets a score strictly higher than GOCat’s. In parenthesis, fraction of publications on which the score is equal to 1 (maximum)

^*d*^Fraction of publications on which GOCat gets a score strictly higher than GOTA’s. In parenthesis, fraction of publications on which the score is equal to 1 (maximum)

Finally, interestingly enough, also with GOCat there is not a dramatic improvement in the classification capabilities when the input query consists of title and abstract (T + A) versus publication title only (T). This is a further confirmation that, independently of the particular approach, titles provide most of the information about the biological content of scientific publications. This may suggest that automated GO annotation tools could benefit from a preprocessing phase aimed at selecting short sentences within the text, in order to filter-out the background noise.

## Conclusions

We described a novel approach for GO annotation of biomedical literature. The resulting annotation tool, GOTA, makes use only of information that is readily available from public repositories and it is easily expandable to handle novel sources of information. The classification capabilities of GOTA are reasonably good in comparison to the state of the art in multi-label classification over large taxonomies and the experimental tests provide some interesting insights into the potential improvement of automated annotation tools. In particular: i) the classification capabilities improve if the approach is tested over specie-specific knowledge bases. This suggests that GO curation task could benefit from developing species-centered annotation tools; ii) the publication title is the most important source of information for GO classification biomedical literature. This result is a strong indication that the biological content of a paper is well-summarized in few short sentences within the text, while the rest is background noise. In this view, the suggestion is that GO annotation tools for literature could benefit from a preprocessing phase aimed at filtering-out background sentences within the text.

## Additional files

Additional file 1
**Benchmark set.** Detailed list of PubMed identifiers and related GO annotations used for the experimental tests. (GZ 2263 kb)

Additional file 2
**Detailed experimental results.** Detailed results of the experimental tests over the three distinct GO categories BP, MF and CC. (PDF 293 kb)

## Abbreviations

GO: Gene ontology
BP: Biological process
MF: Molecular function
CC: Cellular component
DAG: Direct acyclic graph
IR: Information retrieval
k-NN: k-nearest neighbors
KB: Knowledge base
PMID: PubMed identifier
BoW: Bag of words
