# Supplementary material for: GOTA: GO term annotation of biomedical literature
Source: BMC Bioinformatics. 2015 Oct 28;16:346. doi: 10.1186/s12859-015-0777-8 (PMC4625458; doi:10.1186/s12859-015-0777-8)
Supplement: Additional file 2 — Detailed experimental results. Detailed results of the experimental tests over the three distinct GO categories BP, MF and CC. (PDF 293 kb) [file 12859_2015_777_MOESM2_ESM.pdf]

SUPPLEMENTARY MATERIALS
GOTA: GO Term Annotation of biomedical literature

Pietro Di Lena, Giacomo Domeniconi, Luciano Margara, Gianluca Moro

1 Dataset statistics

We randomly divided the entire benchmark corpora, which contains a total of 115,402 publications, in two disjoint sets: a training set of 100,402 documents, which we call knowledge base (KB), and a test set of 15,000 documents. In the following, we compare the distributions of GO terms and references in the three sets: full benchmark set (All), training set (KB) and test set (Test).

The top section of Table 1 shows the percentage of publications that have at least one gold-standard annotation in each one of the three sub-ontologies BP, MF and CC. The bottom section of Table 1 shows the percentage of publications in the three sub-ontologies for which bibliography information is available. As can be seen in Table 1, the three sets are extremely well balanced. Figure 1 and 2 show the distribution and cumulative distribution, respectively, of gold-standard annotations on the entire benchmark set. As can be seen, from Figure 1 and 2, 39% of publications have a single annotations and 99% at most ten district annotations.

Tab. 1: Benchmark set statistics

Dataset (#Docs)	%Docs with BP annot.	%Docs with MF annot.	%Docs with CC annot.
All (115,402)	74.98%	46.54%	35.11%
KB (100,402)	74.98%	46.50%	35.18%
Test (15,000)	74,95%	46.79%	34.68%
Dataset (#Docs)	%Docs with bibl. (BP)	%Docs with bibl. (MF)	%Docs with bibl. (CC)
All (115,402)	40.27%	39.67%	40.86%
KB (100,402)	40.28%	39.64%	40.85%
Test (15,000)	40.21%	39.88%	40.91%

As a further analysis, for each GO term, we computed the difference of the relative frequency of the annotated publications belonging to KB and test set as follows:

$$\text{diff}(t) = \left| \frac{|p \in KB : p \text{ has annotation } t|}{|KB|} - \frac{|p \in Test : p \text{ has annotation } t|}{|Test|} \right|$$

Averaging the diff(t) for each $t \in GO$, the obtained value $AVG_{t \in GO}\text{diff}(t) = 0.005\%$ indicates that the distributions of GO annotations are not very different for the publications in the KB in comparison to those in the test set. We also have an amount of 12,184 GO terms that are represented in the KB and not in the test set. The average number of publications associated to such GO terms is 2.84, which implies that these are very specific terms.

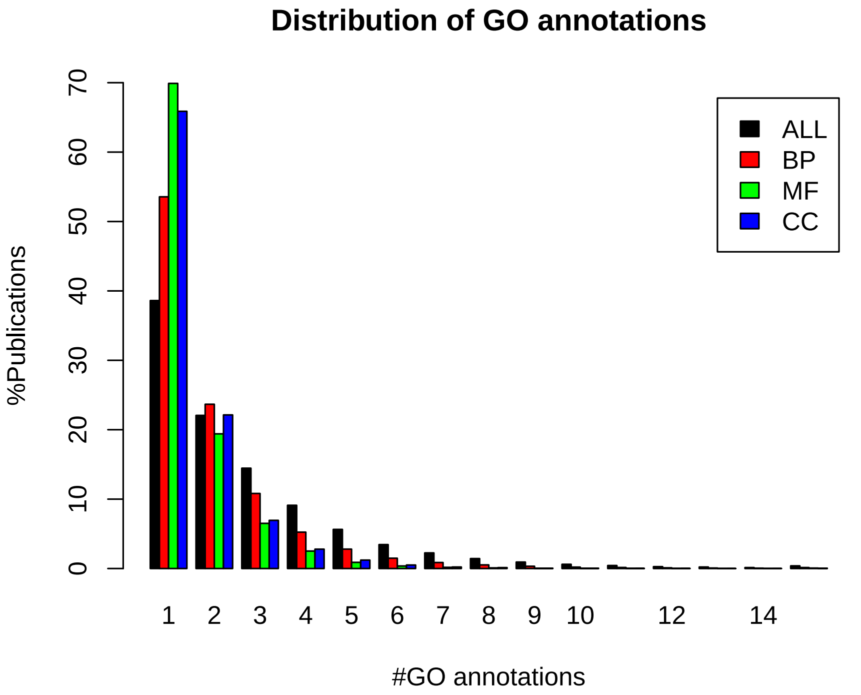

Fig. 1: Distribution of GO annotations over the entire benchmark set

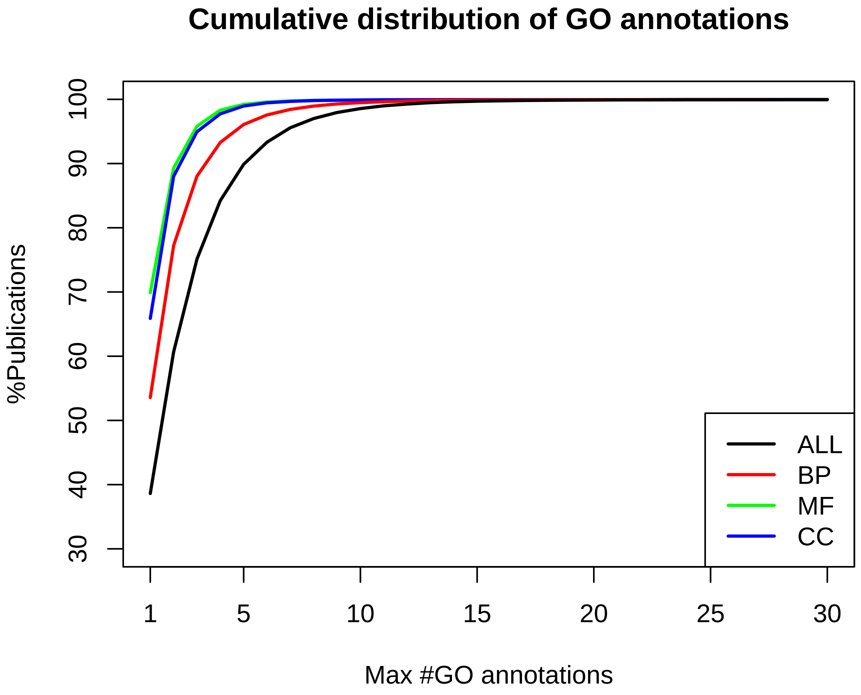

Fig. 2: Cumulative distribution of GO annotations over the entire benchmark set

2 Detailed evaluation results over the three distinct ontologies

We show the detailed classification performances over the entire GO hierarchy and separately over its three main categories Biological Process (BP), Molecular Function (MF) and Cellular Component (CC).

For each category, only the test-publications associated with at least one term in the selected category are considered for performance evaluation (the total number of considered publications is specified for each table). The performances have been assessed for both the top-10 and top-20 predicted terms. For instance, for the evaluation over the BP ontology, exactly the top-k (where, $k = 10$ or $k = 20$) terms of type BP are taken into account. The same holds for the other two categories. In Section 2.3, in order to allow a fair comparison with GOCat over BP, MF and CC, a slightly different evaluation approach has been taken. The web-based GOCat application returns only the top-50 predicted terms; in most of the cases, among the top-50 terms we can observe less than $k = 10$ (or 20) terms of type BP, MF and CC. For this reason, in Section 2.3, for all the considered approaches, we evaluate at most $k = 10$ (or 20) BP, MF and CC terms (possibly none) among the top-50 returned by the classifiers. For completeness and correctness of information, we point out that, with respect to the MRR_{20} metric and over the three distinct ontologies BP,MF and CC, the shown performances of GOCat are not consistent with what reported in (Gobeill *et. al*, Database 2013). In particular, on our test set, we report for GOCat a lower value of MMR_{20} over the full GO and higher values over BP,MF and CC taxonomies.

The evaluation metrics adopted for performance assessment are defined in the "Evaluation metric" Section of the reference paper. We show here also the iP_k, hP_k and the (hierarchical harmonic mean) hF_k metrics. We further include a new evaluation score $\#_k$, which indicates the fraction of publications for which at least one gold-standard annotation has been correctly predicted among the top-k terms returned by the classifier.

For each table and for each metric (excluding hF_{max} and hF_k, which are not averaged) we also show the p-value of the paired student's t-test between GOTA (PM) and the other methods.

2.1 Overall classification performances

2.1.1 Evaluation of the top-10 predicted GO terms

Tab. 2: Performance comparison over all GO (15,000 publications)

Method[a]	Info[b]	IT[c]			CAFA[c]	BC[c]			TREC[c]		$\#^d_{10}$
		iP_1	iR_{10}	iP_{10}	hF_{max}	hF_{10}	hR_{10}	hP_{10}	MRR_{10}	R_{10}	
GOTA	PM	**0.43**	**0.64**	**0.28**	**0.43**	**0.35**	**0.69**	**0.23**	**0.40**	**0.46**	**0.68**
GOTA	T+A	0.42	**0.64**	**0.28**	**0.43**	0.34	0.68	**0.23**	0.39	0.45	0.67
GOTA	T	0.41	0.63	0.27	0.42	0.34	0.68	**0.23**	0.39	0.44	0.67
RandFR	N/A	0.20	0.33	0.15	0.20	0.15	0.33	0.10	0.18	0.15	0.28
RandIC	N/A	0.21	0.27	0.20	0.18	0.17	0.31	0.12	0.03	0.08	0.14
GOTA Φ_P	PM	0.37	**0.64**	0.26	0.41	0.33	0.67	0.22	0.38	0.44	0.66
GOTA Φ_P	T+A	0.35	0.62	0.25	0.40	0.33	0.66	0.22	0.36	0.41	0.63
GOTA Φ_P	T	0.35	0.62	0.25	0.40	0.33	0.66	0.22	0.36	0.41	0.63
GOTA Φ_T	PM	0.28	0.41	0.22	0.30	0.26	0.49	0.18	0.16	0.17	0.32
GOTA Φ_T	T+A	0.24	0.37	0.20	0.27	0.24	0.46	0.16	0.11	0.12	0.23
GOTA Φ_T	T	0.22	0.35	0.19	0.26	0.22	0.44	0.15	0.09	0.10	0.19

For each metric, the best result is highlighted in bold.

Tab. 3: Statistical significance (p-value) of the paired t-test between GOTA (PM) and the other methods over all GO (15,000 publications)

Method[a]	Info[b]	iP_1	iR_{10}	iP_{10}	hR_{10}	hP_{10}	MRR_{10}	R_{10}	$\#_{10}$
GOTA	T+A	3.030e-09	0.0006598	<2.2e-16	2.205e-13	0.00301	4.957e-14	1.150e-13	2.331e-09
GOTA	T	5.849e-14	1.948e-10	<2.2e-16	<2.2e-16	3.144e-11	<2.2e-16	<2.2e-16	<2.2e-16
RandFR	N/A	<2.2e-16	<2.2e-16	<2.2e-16	<2.2e-16	<2.2e-16	<2.2e-16	<2.2e-16	<2.2e-16
RandIC	N/A	<2.2e-16	<2.2e-16	<2.2e-16	<2.2e-16	<2.2e-16	<2.2e-16	<2.2e-16	<2.2e-16
GOTA Φ_P	PM	<2.2e-16	0.00386	<2.2e-16	<2.2e-16	2.221e-11	<2.2e-16	<2.2e-16	<2.2e-16
GOTA Φ_P	T+A	<2.2e-16	<2.2e-16	<2.2e-16	<2.2e-16	<2.2e-16	<2.2e-16	<2.2e-16	<2.2e-16
GOTA Φ_P	T	<2.2e-16	<2.2e-16	<2.2e-16	<2.2e-16	<2.2e-16	<2.2e-16	<2.2e-16	<2.2e-16
GOTA Φ_T	PM	<2.2e-16	<2.2e-16	<2.2e-16	<2.2e-16	<2.2e-16	<2.2e-16	<2.2e-16	<2.2e-16
GOTA Φ_T	T+A	<2.2e-16	<2.2e-16	<2.2e-16	<2.2e-16	<2.2e-16	<2.2e-16	<2.2e-16	<2.2e-16
GOTA Φ_T	T	<2.2e-16	<2.2e-16	<2.2e-16	<2.2e-16	<2.2e-16	<2.2e-16	<2.2e-16	<2.2e-16

Not significant p-values are highlighted in bold.

Tab. 4: Performance comparison over BP (11,242 publications)

Method[a]	Info[b]	IT[c]			CAFA[c]	BC[c]			TREC[c]		$\#_{10}^{d}$
		iP_1	iR_{10}	iP_{10}	hF_{max}	hF_{10}	hR_{10}	hP_{10}	MRR_{10}	R_{10}	
GOTA	PM	**0.50**	**0.72**	**0.34**	**0.50**	**0.34**	**0.76**	**0.22**	**0.38**	**0.49**	**0.64**
GOTA	T+A	0.49	0.71	**0.34**	0.49	**0.34**	0.75	**0.22**	0.36	0.47	0.62
GOTA	T	0.49	0.70	**0.34**	0.49	**0.34**	0.75	**0.22**	0.36	0.47	0.62
RandFR	N/A	0.21	0.35	0.20	0.17	0.16	0.35	0.10	0.04	0.06	0.10
RandIC	N/A	0.29	0.32	0.26	0.26	0.20	0.36	0.14	0.01	0.01	0.02
GOTA Φ_P	PM	0.48	0.71	0.33	0.48	0.33	0.75	0.21	0.36	0.46	0.61
GOTA Φ_P	T+A	0.47	0.69	0.33	0.47	0.33	0.73	0.21	0.33	0.44	0.59
GOTA Φ_P	T	0.47	0.69	0.33	0.47	0.33	0.74	0.21	0.33	0.44	0.59
GOTA Φ_T	PM	0.33	0.52	0.26	0.37	0.29	0.60	0.19	0.15	0.21	0.31
GOTA Φ_T	T+A	0.29	0.48	0.24	0.34	0.26	0.57	0.17	0.11	0.15	0.23
GOTA Φ_T	T	0.27	0.46	0.24	0.32	0.25	0.55	0.16	0.09	0.13	0.19

For each metric, the best result is highlighted in bold.

Tab. 5: Statistical significance (p-value) of the paired t-test between GOTA (PM) and the other methods over BP (11,242 publications)

Method[a]	Info[b]	iP_1	iR_{10}	iP_{10}	hR_{10}	hP_{10}	MRR_{10}	R_{10}	$\#_{10}$
GOTA	T+A	8.228e-09	1.786e-10	**0.05258**	4.567e-12	2.485e-07	<2.2e-16	<2.2e-16	1.158e-13
GOTA	T	6.619e-13	<2.2e-16	6.491e-08	<2.2e-16	<2.2e-16	<2.2e-16	<2.2e-16	<2.2e-16
RandFR	N/A	<2.2e-16	<2.2e-16	<2.2e-16	<2.2e-16	<2.2e-16	<2.2e-16	<2.2e-16	<2.2e-16
RandIC	N/A	<2.2e-16	<2.2e-16	<2.2e-16	<2.2e-16	<2.2e-16	<2.2e-16	<2.2e-16	<2.2e-16
GOTA Φ_P	PM	4.623e-11	4.161e-07	<2.2e-16	<2.2e-16	<2.2e-16	<2.2e-16	<2.2e-16	<2.2e-16
GOTA Φ_P	T+A	<2.2e-16	<2.2e-16	<2.2e-16	<2.2e-16	<2.2e-16	<2.2e-16	<2.2e-16	<2.2e-16
GOTA Φ_P	T	<2.2e-16	<2.2e-16	<2.2e-16	<2.2e-16	<2.2e-16	<2.2e-16	<2.2e-16	<2.2e-16
GOTA Φ_T	PM	<2.2e-16	<2.2e-16	<2.2e-16	<2.2e-16	<2.2e-16	<2.2e-16	<2.2e-16	<2.2e-16
GOTA Φ_T	T+A	<2.2e-16	<2.2e-16	<2.2e-16	<2.2e-16	<2.2e-16	<2.2e-16	<2.2e-16	<2.2e-16
GOTA Φ_T	T	<2.2e-16	<2.2e-16	<2.2e-16	<2.2e-16	<2.2e-16	<2.2e-16	<2.2e-16	<2.2e-16

Not significant p-values are highlighted in bold.

Tab. 6: Performance comparison over MF (7,019 publications)

Method[a]	Info[b]	IT[c]			CAFA[c]	BC[c]			TREC[c]		$\#_{10}^{d}$
		iP_1	iR_{10}	iP_{10}	hF_{max}	hF_{10}	hR_{10}	hP_{10}	MRR_{10}	R_{10}	
GOTA	PM	**0.60**	**0.75**	**0.35**	**0.65**	0.33	**0.88**	0.20	**0.54**	**0.71**	**0.79**
GOTA	T+A	0.58	0.74	**0.35**	0.64	0.33	**0.88**	0.20	0.52	0.70	0.78
GOTA	T	0.57	0.74	**0.35**	0.64	0.33	0.87	0.20	0.52	0.69	0.77
RandFR	N/A	0.43	0.46	0.27	0.49	0.17	0.59	0.10	0.31	0.32	0.39
RandIC	N/A	0.43	0.45	0.32	0.49	**0.35**	0.50	**0.27**	0.28	0.26	0.32
GOTA Φ_P	PM	0.55	**0.75**	0.34	**0.65**	0.31	**0.88**	0.19	0.52	0.69	0.77
GOTA Φ_P	T+A	0.53	0.74	0.33	0.62	0.31	0.86	0.19	0.49	0.67	0.75
GOTA Φ_P	T	0.53	0.74	0.34	0.63	0.31	0.87	0.19	0.49	0.67	0.75
GOTA Φ_T	PM	0.38	0.56	0.30	0.49	0.29	0.75	0.18	0.18	0.27	0.33
GOTA Φ_T	T+A	0.34	0.52	0.28	0.46	0.28	0.73	0.17	0.13	0.21	0.26
GOTA Φ_T	T	0.33	0.51	0.28	0.45	0.27	0.71	0.17	0.11	0.20	0.24

For each metric, the best result is highlighted in bold.

Tab. 7: Statistical significance (p-value) of the paired t-test between GOTA (PM) and the other methods over MF (7,019 publications)

Method[a]	Info[b]	iP_1	iR_{10}	iP_{10}	hR_{10}	hP_{10}	MRR_{10}	R_{10}	$\#_{10}$
GOTA	T+A	1.399e-14	0.002341	1.898e-10	6.625e-06	2.053e-07	7.894e-13	3.545e-08	1.056e-06
GOTA	T	1.051e-15	8.118e-06	8.268e-15	1.086e-09	1.601e-13	3.447e-14	1.395e-12	4.949e-10
RandFR	N/A	<2.2e-16	<2.2e-16	<2.2e-16	<2.2e-16	<2.2e-16	<2.2e-16	<2.2e-16	<2.2e-16
RandIC	N/A	<2.2e-16	<2.2e-16	<2.2e-16	<2.2e-16	**1**	<2.2e-16	<2.2e-16	<2.2e-16
GOTA Φ_P	PM	<2.2e-16	**0.6325**	<2.2e-16	5.347e-07	<2.2e-16	4.347e-11	2.652e-11	2.18e-12
GOTA Φ_P	T+A	<2.2e-16	2.714e-05	<2.2e-16	<2.2e-16	<2.2e-16	<2.2e-16	<2.2e-16	<2.2e-16
GOTA Φ_P	T	<2.2e-16	5.719e-05	<2.2e-16	<2.2e-16	<2.2e-16	<2.2e-16	<2.2e-16	<2.2e-16
GOTA Φ_T	PM	<2.2e-16	<2.2e-16	<2.2e-16	<2.2e-16	<2.2e-16	<2.2e-16	<2.2e-16	<2.2e-16
GOTA Φ_T	T+A	<2.2e-16	<2.2e-16	<2.2e-16	<2.2e-16	<2.2e-16	<2.2e-16	<2.2e-16	<2.2e-16
GOTA Φ_T	T	<2.2e-16	<2.2e-16	<2.2e-16	<2.2e-16	<2.2e-16	<2.2e-16	<2.2e-16	<2.2e-16

Not significant p-values are highlighted in bold.

Tab. 8: Performance comparison over CC (5,202 publications)

Method[a]	Info[b]	IT[c]			CAFA[c]	BC[c]			TREC[c]		$\#^d_{10}$
		iP_1	iR_{10}	iP_{10}	hF_{max}	hF_{10}	hR_{10}	hP_{10}	MRR_{10}	R_{10}	
GOTA	PM	**0.62**	**0.86**	0.42	**0.64**	0.40	**0.90**	0.26	**0.52**	**0.73**	**0.81**
GOTA	T+A	0.61	0.85	0.42	**0.64**	0.39	**0.90**	0.25	0.51	0.71	0.80
GOTA	T	0.61	0.85	0.42	0.63	0.39	**0.90**	0.25	0.50	0.71	0.80
RandFR	N/A	0.50	0.68	0.37	0.52	0.38	0.71	0.26	0.30	0.48	0.56
RandIC	N/A	0.50	0.56	**0.46**	0.53	**0.50**	0.60	**0.43**	0.12	0.23	0.30
GOTA Φ_P	PM	0.61	**0.86**	0.42	**0.64**	0.40	**0.90**	0.26	0.51	0.72	0.80
GOTA Φ_P	T+A	0.60	**0.86**	0.42	0.62	0.40	0.89	0.26	0.50	0.71	0.79
GOTA Φ_P	T	0.60	**0.86**	0.42	0.62	0.40	0.89	0.26	0.50	0.71	0.79
GOTA Φ_T	PM	0.37	0.49	0.31	0.46	0.31	0.66	0.20	0.15	0.21	0.27
GOTA Φ_T	T+A	0.35	0.47	0.29	0.45	0.30	0.64	0.20	0.12	0.18	0.23
GOTA Φ_T	T	0.33	0.44	0.28	0.44	0.30	0.62	0.20	0.09	0.14	0.19

For each metric, the best result is highlighted in bold.

Tab. 9: Statistical significance (p-value) of the paired t-test between GOTA (PM) and the other methods over CC (5,202 publications)

Method[a]	Info[b]	iP_1	iR_{10}	iP_{10}	hR_{10}	hP_{10}	MRR_{10}	R_{10}	$\#_{10}$
GOTA	T+A	0.02054	2.198e-06	9.493e-10	4.805e-05	0.0003024	8.635e-05	1.233e-06	1.892e-06
GOTA	T	0.0002178	2.339e-09	1.881e-14	3.897e-08	9.646e-10	2.325e-08	2.904e-09	1.531e-08
RandFR	N/A	<2.2e-16	<2.2e-16	<2.2e-16	<2.2e-16	**0.9997**	<2.2e-16	<2.2e-16	<2.2e-16
RandIC	N/A	<2.2e-16	<2.2e-16	**1**	<2.2e-16	**1**	<2.2e-16	<2.2e-16	<2.2e-16
GOTA Φ_P	PM	**0.07303**	**0.4768**	0.0006042	0.0004766	**0.9999**	**0.05582**	0.0003123	8.63e-05
GOTA Φ_P	T+A	9.323e-07	0.004878	6.046e-07	2.252e-08	**0.772**	3.452e-10	1.839e-09	5.675e-09
GOTA Φ_P	T	1.543e-06	0.003809	4.949e-07	1.673e-08	**0.7736**	8.228e-10	3.562e-09	1.091e-08
GOTA Φ_T	PM	<2.2e-16	<2.2e-16	<2.2e-16	<2.2e-16	<2.2e-16	<2.2e-16	<2.2e-16	<2.2e-16
GOTA Φ_T	T+A	<2.2e-16	<2.2e-16	<2.2e-16	<2.2e-16	<2.2e-16	<2.2e-16	<2.2e-16	<2.2e-16
GOTA Φ_T	T	<2.2e-16	<2.2e-16	<2.2e-16	<2.2e-16	<2.2e-16	<2.2e-16	<2.2e-16	<2.2e-16

Not significant p-values are highlighted in bold.

[a] Method used for prediction. RandFR and RandIC are baseline predictors.

[b] Informations used in prediction: PM = title, abstract, references and publication year (PubMed); T+A = title and abstract; T = title; N/A = no information.

[c] Metrics definitions are in Section 2.3.

[d] Fraction of publications for which at least one gold-standard annotation has been correctly predicted.

2.1.2 Evaluation of the top-20 predicted GO terms

Tab. 10: Performance comparison over all GO (15,000 publications)

Method[a]	Info[b]	IT[c]			CAFA[c]	BC[c]			TREC[c]		$\#^d_{20}$
		iP_1	iR_{20}	iP_{20}	hF_{max}	hF_{20}	hR_{20}	hP_{20}	MRR_{20}	R_{20}	
GOTA	PM	**0.43**	**0.73**	**0.24**	**0.43**	**0.27**	**0.80**	**0.16**	**0.41**	**0.56**	**0.77**
GOTA	T+A	0.42	0.72	**0.24**	**0.43**	**0.27**	0.79	**0.16**	0.40	0.55	0.76
GOTA	T	0.41	0.72	**0.24**	0.42	**0.27**	0.78	**0.16**	0.39	0.54	0.75
RandFR	N/A	0.20	0.40	0.14	0.20	0.15	0.43	0.09	0.18	0.19	0.34
RandIC	N/A	0.21	0.31	0.19	0.18	0.10	0.38	0.06	0.03	0.08	0.15
GOTA Φ_P	PM	0.37	**0.73**	0.23	0.41	**0.27**	0.78	**0.16**	0.39	0.54	0.75
GOTA Φ_P	T+A	0.35	0.71	0.22	0.40	0.25	0.77	0.15	0.37	0.52	0.73
GOTA Φ_P	T	0.35	0.71	0.22	0.40	0.25	0.77	0.15	0.37	0.52	0.73
GOTA Φ_T	PM	0.28	0.48	0.20	0.30	0.21	0.58	0.13	0.16	0.22	0.40
GOTA Φ_T	T+A	0.24	0.45	0.19	0.27	0.20	0.56	0.12	0.11	0.17	0.30
GOTA Φ_T	T	0.22	0.42	0.18	0.26	0.18	0.54	0.11	0.09	0.14	0.27

For each metric, the best result is highlighted in bold.

Tab. 11: Statistical significance (p-value) of the paired t-test between GOTA (PM) and the other methods over all GO (15,000 publications)

Method[a]	Info[b]	iP_1	iR_{20}	iP_{20}	hR_{20}	hP_{20}	MRR_{20}	R_{20}	$\#^d_{20}$
GOTA	T+A	3.030e-09	4.768e-08	6.664e-15	6.705e-14	0.000633	1.529e-14	<2.2e-16	6.064e-14
GOTA	T	5.849e-14	<2.2e-16	<2.2e-16	<2.2e-16	2.392e-13	<2.2e-16	<2.2e-16	<2.2e-16
RandFR	N/A	<2.2e-16	<2.2e-16	<2.2e-16	<2.2e-16	<2.2e-16	<2.2e-16	<2.2e-16	<2.2e-16
RandIC	N/A	<2.2e-16	<2.2e-16	<2.2e-16	<2.2e-16	<2.2e-16	<2.2e-16	<2.2e-16	<2.2e-16
GOTA Φ_P	PM	<2.2e-16	0.002566	<2.2e-16	<2.2e-16	<2.2e-16	<2.2e-16	<2.2e-16	<2.2e-16
GOTA Φ_P	T+A	<2.2e-16	<2.2e-16	<2.2e-16	<2.2e-16	<2.2e-16	<2.2e-16	<2.2e-16	<2.2e-16
GOTA Φ_P	T	<2.2e-16	<2.2e-16	<2.2e-16	<2.2e-16	<2.2e-16	<2.2e-16	<2.2e-16	<2.2e-16
GOTA Φ_T	PM	<2.2e-16	<2.2e-16	<2.2e-16	<2.2e-16	<2.2e-16	<2.2e-16	<2.2e-16	<2.2e-16
GOTA Φ_T	T+A	<2.2e-16	<2.2e-16	<2.2e-16	<2.2e-16	<2.2e-16	<2.2e-16	<2.2e-16	<2.2e-16
GOTA Φ_T	T	<2.2e-16	<2.2e-16	<2.2e-16	<2.2e-16	<2.2e-16	<2.2e-16	<2.2e-16	<2.2e-16

Not significant p-values are highlighted in bold.

Tab. 12: Performance comparison over BP (11,242 publications)

Method[a]	Info[b]	IT[c]			CAFA[c]	BC[c]			TREC[c]		$\#_{20}^{d}$
		iP_1	iR_{20}	iP_{20}	hF_{max}	hF_{20}	hR_{20}	hP_{20}	MRR_{20}	R_{20}	
GOTA	PM	**0.50**	**0.78**	**0.30**	**0.50**	**0.24**	**0.84**	**0.14**	**0.39**	**0.58**	**0.72**
GOTA	T+A	0.49	**0.78**	**0.30**	0.49	**0.24**	0.83	**0.14**	0.37	0.56	0.70
GOTA	T	0.49	0.77	0.29	0.49	**0.24**	0.83	**0.14**	0.37	0.56	0.70
RandFR	N/A	0.21	0.43	0.17	0.17	0.15	0.48	0.09	0.04	0.10	0.15
RandIC	N/A	0.29	0.36	0.25	0.26	0.12	0.47	0.07	0.01	0.02	0.03
GOTA Φ_P	PM	0.48	**0.78**	0.28	0.48	**0.24**	0.83	**0.14**	0.36	0.55	0.70
GOTA Φ_P	T+A	0.47	0.77	0.28	0.47	**0.24**	0.82	**0.14**	0.34	0.53	0.67
GOTA Φ_P	T	0.47	0.77	0.28	0.47	**0.24**	0.82	**0.14**	0.34	0.53	0.67
GOTA Φ_T	PM	0.33	0.59	0.25	0.37	0.22	0.68	0.13	0.16	0.28	0.39
GOTA Φ_T	T+A	0.29	0.55	0.23	0.34	0.22	0.65	0.13	0.11	0.21	0.31
GOTA Φ_T	T	0.27	0.53	0.22	0.32	0.20	0.64	0.12	0.09	0.18	0.27

For each metric, the best result is highlighted in bold.

Tab. 13: Statistical significance (p-value) of the paired t-test between GOTA (PM) and the other methods over BP (11,242 publications)

Method[a]	Info[b]	iP_1	iR_{20}	iP_{20}	hR_{20}	hP_{20}	MRR_{20}	R_{20}	$\#_{20}^{d}$
GOTA	T+A	8.228e-09	5.778e-08	**0.8528**	6.887e-11	0.01363	<2.2e-16	<2.2e-16	1.039e-11
GOTA	T	6.619e-13	<2.2e-16	0.0001168	<2.2e-16	9.995e-12	<2.2e-16	<2.2e-16	<2.2e-16
RandFR	N/A	<2.2e-16	<2.2e-16	<2.2e-16	<2.2e-16	<2.2e-16	<2.2e-16	<2.2e-16	<2.2e-16
RandIC	N/A	<2.2e-16	<2.2e-16	<2.2e-16	<2.2e-16	<2.2e-16	<2.2e-16	<2.2e-16	<2.2e-16
GOTA Φ_P	PM	4.623e-11	0.0002218	<2.2e-16	<2.2e-16	<2.2e-16	<2.2e-16	<2.2e-16	<2.2e-16
GOTA Φ_P	T+A	<2.2e-16	<2.2e-16	<2.2e-16	<2.2e-16	<2.2e-16	<2.2e-16	<2.2e-16	<2.2e-16
GOTA Φ_P	T	<2.2e-16	<2.2e-16	<2.2e-16	<2.2e-16	<2.2e-16	<2.2e-16	<2.2e-16	<2.2e-16
GOTA Φ_T	PM	<2.2e-16	<2.2e-16	<2.2e-16	<2.2e-16	<2.2e-16	<2.2e-16	<2.2e-16	<2.2e-16
GOTA Φ_T	T+A	<2.2e-16	<2.2e-16	<2.2e-16	<2.2e-16	<2.2e-16	<2.2e-16	<2.2e-16	<2.2e-16
GOTA Φ_T	T	<2.2e-16	<2.2e-16	<2.2e-16	<2.2e-16	<2.2e-16	<2.2e-16	<2.2e-16	<2.2e-16

Not significant p-values are highlighted in bold.

Tab. 14: Performance comparison over MF (7,019 publications)

Method[a]	Info[b]	IT[c]			CAFA[c]	BC[c]			TREC[c]		$\#_{20}^{d}$
		iP_1	iR_{20}	iP_{20}	hF_{max}	hF_{20}	hR_{20}	hP_{20}	MRR_{20}	R_{20}	
GOTA	PM	**0.60**	**0.78**	**0.31**	**0.65**	0.21	**0.92**	0.12	**0.54**	**0.77**	**0.84**
GOTA	T+A	0.58	0.77	0.30	0.64	0.21	**0.92**	0.12	0.53	0.76	0.83
GOTA	T	0.57	0.77	0.30	0.64	0.21	**0.92**	0.12	0.53	0.76	0.82
RandFR	N/A	0.43	0.53	0.26	0.49	0.13	0.64	0.07	0.31	0.38	0.45
RandIC	N/A	0.43	0.50	0.30	0.49	**0.24**	0.56	**0.15**	0.29	0.29	0.36
GOTA Φ_P	PM	0.55	**0.78**	0.30	**0.65**	0.20	**0.92**	0.11	0.52	0.76	0.83
GOTA Φ_P	T+A	0.53	**0.78**	0.29	0.63	0.20	0.91	0.11	0.49	0.75	0.81
GOTA Φ_P	T	0.53	**0.78**	0.29	0.63	0.20	0.91	0.11	0.49	0.75	0.81
GOTA Φ_T	PM	0.38	0.61	0.28	0.49	0.21	0.80	0.12	0.18	0.33	0.40
GOTA Φ_T	T+A	0.34	0.58	0.27	0.46	0.21	0.78	0.12	0.13	0.27	0.33
GOTA Φ_T	T	0.33	0.56	0.27	0.45	0.21	0.77	0.12	0.12	0.25	0.31

For each metric, the best result is highlighted in bold.

Tab. 15: Statistical significance (p-value) of the paired t-test between GOTA (PM) and the other methods over MF (7,019 publications)

Method[a]	Info[b]	iP_1	iR_{20}	iP_{20}	hR_{20}	hP_{20}	MRR_{20}	R_{20}	$\#_{20}^{d}$
GOTA	T+A	1.399e-14	0.0006626	1.103e-15	4.528e-05	3.163e-08	6.99e-13	1.953e-08	5.146e-07
GOTA	T	1.051e-15	1.127e-05	<2.2e-16	6.694e-08	8.245e-15	3.727e-14	4.83e-12	8.111e-10
RandFR	N/A	<2.2e-16	<2.2e-16	<2.2e-16	<2.2e-16	<2.2e-16	<2.2e-16	<2.2e-16	<2.2e-16
RandIC	N/A	<2.2e-16	<2.2e-16	0.0004285	<2.2e-16	**1**	<2.2e-16	<2.2e-16	<2.2e-16
GOTA Φ_P	PM	<2.2e-16	**0.9657**	<2.2e-16	4.858e-06	<2.2e-16	9.48e-11	3.091e-11	2.762e-09
GOTA Φ_P	T+A	<2.2e-16	0.006705	<2.2e-16	1.58e-15	<2.2e-16	<2.2e-16	<2.2e-16	<2.2e-16
GOTA Φ_P	T	<2.2e-16	0.007871	<2.2e-16	3.156e-15	<2.2e-16	<2.2e-16	<2.2e-16	<2.2e-16
GOTA Φ_T	PM	<2.2e-16	<2.2e-16	<2.2e-16	<2.2e-16	**0.7374**	<2.2e-16	<2.2e-16	<2.2e-16
GOTA Φ_T	T+A	<2.2e-16	<2.2e-16	<2.2e-16	<2.2e-16	**0.0771**	<2.2e-16	<2.2e-16	<2.2e-16
GOTA Φ_T	T	<2.2e-16	<2.2e-16	<2.2e-16	<2.2e-16	0.001684	<2.2e-16	<2.2e-16	<2.2e-16

Not significant p-values are highlighted in bold.

Tab. 16: Performance comparison over CC (5,202 publications)

Method[a]	Info[b]	IT[c]			CAFA[c]	BC[c]			TREC[c]		$\#_{20}^{d}$
		iP_1	iR_{20}	iP_{20}	hF_{max}	hF_{20}	hR_{20}	hP_{20}	MRR_{20}	R_{20}	
GOTA	PM	**0.62**	**0.90**	0.38	**0.64**	0.27	**0.94**	0.16	**0.52**	**0.81**	**0.88**
GOTA	T+A	0.61	0.89	0.37	**0.64**	0.27	**0.94**	0.16	**0.52**	0.80	0.87
GOTA	T	0.61	0.89	0.37	0.63	0.27	**0.94**	0.16	0.51	0.80	0.86
RandFR	N/A	0.50	0.77	0.34	0.52	0.25	0.80	0.15	0.31	0.57	0.65
RandIC	N/A	0.50	0.66	**0.41**	0.53	**0.32**	0.71	**0.21**	0.13	0.38	0.46
GOTA Φ_P	PM	0.61	**0.90**	0.37	**0.64**	0.27	**0.94**	0.16	**0.52**	0.80	0.87
GOTA Φ_P	T+A	0.60	**0.90**	0.37	0.62	0.27	**0.94**	0.16	0.50	0.79	0.86
GOTA Φ_P	T	0.60	**0.90**	0.37	0.62	0.27	**0.94**	0.16	0.50	0.79	0.86
GOTA Φ_T	PM	0.37	0.53	0.29	0.46	0.23	0.71	0.14	0.16	0.25	0.32
GOTA Φ_T	T+A	0.35	0.51	0.28	0.45	0.23	0.69	0.14	0.12	0.23	0.29
GOTA Φ_T	T	0.33	0.48	0.27	0.44	0.23	0.67	0.14	0.10	0.18	0.24

For each metric, the best result is highlighted in bold.

Tab. 17: Statistical significance (p-value) of the paired t-test between GOTA (PM) and the other methods over CC (5,202 publications)

Method[a]	Info[b]	iP_1	iR_{20}	iP_{20}	hR_{20}	hP_{20}	MRR_{20}	R_{20}	$\#_{20}^{d}$
GOTA	T+A	0.02054	1.064e-06	6.61e-15	3.274e-05	6.687e-06	0.0001070	3.488e-09	3.918e-06
GOTA	T	0.0002178	6.3e-08	<2.2e-16	2.983e-06	3.865e-12	3.626e-08	1.896e-10	7.461e-07
RandFR	N/A	<2.2e-16	<2.2e-16	<2.2e-16	<2.2e-16	<2.2e-16	<2.2e-16	<2.2e-16	<2.2e-16
RandIC	N/A	<2.2e-16	<2.2e-16	**1**	<2.2e-16	**1**	<2.2e-16	<2.2e-16	<2.2e-16
GOTA Φ_P	PM	**0.07303**	**0.3083**	5.809e-11	0.0001568	0.007144	**0.06104**	3.689e-07	1.864e-05
GOTA Φ_P	T+A	9.323e-07	0.0004821	2.736e-15	1.051e-08	0.003394	4.633e-10	4.253e-13	1.053e-08
GOTA Φ_P	T	1.543e-06	0.0007846	9.242e-15	5.228e-08	0.005091	1.141e-09	3.690e-12	4.386e-08
GOTA Φ_T	PM	<2.2e-16	<2.2e-16	<2.2e-16	<2.2e-16	<2.2e-16	<2.2e-16	<2.2e-16	<2.2e-16
GOTA Φ_T	T+A	<2.2e-16	<2.2e-16	<2.2e-16	<2.2e-16	<2.2e-16	<2.2e-16	<2.2e-16	<2.2e-16
GOTA Φ_T	T	<2.2e-16	<2.2e-16	<2.2e-16	<2.2e-16	<2.2e-16	<2.2e-16	<2.2e-16	<2.2e-16

Not significant p-values are highlighted in bold.

[a] Method used for prediction. RandFR and RandIC are baseline predictors.

[b] Informations used in prediction: PM = title, abstract, references and publication year (PubMed); T+A = title and abstract; T = title; N/A = no information.

[c] Metrics definitions are in Section 2.3.

[d] Fraction of publications for which at least one gold-standard annotation has been correctly predicted.

2.2 Performances on species-specific knowledge bases

2.2.1 Evaluation of the top-10 predicted GO terms

Tab. 18: Performance comparison over all GO

Species[a]	KB[b]	Info[c]	IT[d]			CAFA[d]	BC[d]			TREC[d]		#[e]
			iP_1	iR_{10}	iP_{10}	hF_{max}	hF_{10}	hR_{10}	hP_{10}	MRR_{10}	R_{10}	
Human	Human	PM	**0.45**	**0.62**	0.28	**0.46**	**0.36**	**0.69**	**0.24**	**0.49**	**0.49**	**0.75**
Human	Full	PM	0.44	**0.62**	**0.29**	0.44	**0.36**	**0.69**	**0.24**	0.44	0.48	0.74
Human	Human	T	0.42	0.60	0.28	0.45	0.34	0.66	0.23	0.46	0.47	0.72
Human	Full	T	0.44	0.61	**0.29**	0.44	0.35	0.68	**0.24**	0.45	0.47	0.73
Mouse	Mouse	PM	**0.45**	**0.63**	**0.31**	**0.45**	0.36	**0.67**	0.25	**0.45**	**0.44**	**0.75**
Mouse	Full	PM	**0.45**	0.61	**0.31**	0.44	**0.37**	0.66	**0.26**	0.43	0.42	0.73
Mouse	Mouse	T	0.42	**0.63**	0.30	0.44	0.36	0.65	0.25	0.43	0.42	0.73
Mouse	Full	T	0.44	0.60	0.30	0.43	**0.37**	0.64	**0.26**	0.42	0.41	0.71
Rat	Rat	PM	**0.38**	**0.64**	**0.23**	**0.41**	**0.29**	**0.69**	**0.18**	**0.36**	**0.44**	**0.60**
Rat	Full	PM	0.34	0.61	0.22	0.37	0.28	0.67	**0.18**	0.33	0.42	0.58
Rat	Rat	T	0.37	0.62	**0.23**	0.40	0.28	0.67	**0.18**	0.34	0.42	0.58
Rat	Full	T	0.33	0.61	0.22	0.37	0.28	0.66	**0.18**	0.33	0.42	0.58
Yeast	Yeast	PM	**0.45**	**0.72**	0.29	**0.47**	0.35	**0.77**	0.23	**0.42**	**0.50**	**0.67**
Yeast	Full	PM	0.43	0.70	**0.30**	**0.47**	**0.38**	0.75	**0.25**	0.39	0.49	0.66
Yeast	Yeast	T	0.41	0.68	0.28	0.44	0.34	0.74	0.22	0.37	0.45	0.63
Yeast	Full	T	0.41	0.68	0.28	0.44	0.37	0.73	**0.25**	0.35	0.46	0.63

[a] Human: 3,575 publications; Mouse: 2,825 publications; Rat: 2,380 publications; Yeast: 1,290 publications.

Tab. 19: Statistical significance (p-value) of the paired t-test between GOTA (PM, species-specific KB) and the other methods over all GO

Species[a]	KB[b]	Info[c]	iP_1	iR_{10}	iP_{10}	hR_{10}	hP_{10}	MRR_{10}	R_{10}	$\#^e_{10}$
Human	Full	PM	0.02073	**0.1420**	**1**	**0.2781**	**0.8766**	<2.2e-16	0.003281	**0.1332**
Human	Human	T	1.659e-12	2.589e-08	3.655e-11	<2.2e-16	6.267e-05	<2.2e-16	<2.2e-16	9.368e-12
Human	Full	T	0.008581	**0.05796**	**0.9785**	0.0001679	**0.2813**	<2.2e-16	1.163e-07	0.002301
Mouse	Full	PM	**0.568**	5.244e-14	**0.9654**	1.191e-06	**0.9996**	5.195e-06	0.0001971	0.002021
Mouse	Mouse	T	3.392e-07	0.0004156	0.001139	1.963e-10	0.003978	1.155e-09	1.153e-09	3.022e-05
Mouse	Full	T	0.02507	<2.2e-16	0.009992	7.172e-16	**0.9551**	2.278e-09	9.867e-11	3.799e-08
Rat	Full	PM	2.692e-09	2.411e-07	0.004839	2.166e-05	0.006462	1.289e-07	0.001132	0.01125
Rat	Rat	T	0.04483	5.87e-05	0.000701	2.559e-10	**0.1558**	4.177e-08	7.902e-07	3.533e-06
Rat	Full	T	1.285e-10	9.218e-08	4.207e-05	2.119e-08	0.0002506	3.27e-08	0.0001369	0.006473
Yeast	Full	PM	0.00985	0.000915	**0.9862**	0.0001679	**1**	0.0001380	0.03849	**0.1604**
Yeast	Yeast	T	4.792e-07	2.112e-13	5.497e-06	8.782e-12	2.522e-08	2.562e-16	3.364e-14	2.854e-08
Yeast	Full	T	8.055e-07	5.092e-08	0.02096	6.917e-10	**1**	4.528e-16	1.020e-06	9.152e-05

Not significant p-values are highlighted in bold.

[a] Human: 3,575 publications; Mouse: 2,825 publications; Rat: 2,380 publications; Yeast: 1,290 publications.

Tab. 20: Performance comparison over BP

Species[a]	KB[b]	Info[c]	IT[d]			CAFA[d]	BC[d]			TREC[d]		#[e]
			iP_1	iR_{10}	iP_{10}	hF_{max}	hF_{10}	hR_{10}	hP_{10}	MRR_{10}	R_{10}	
Human	Human	PM	**0.49**	0.69	0.34	**0.48**	0.34	**0.74**	0.22	**0.36**	**0.45**	0.61
Human	Full	PM	0.48	**0.70**	**0.35**	**0.48**	**0.35**	**0.74**	**0.23**	0.35	**0.45**	**0.62**
Human	Human	T	0.47	0.67	0.33	0.47	0.34	0.71	0.22	0.33	0.42	0.58
Human	Full	T	0.47	0.68	**0.35**	0.47	**0.35**	0.73	**0.23**	0.33	0.43	0.60
Mouse	Mouse	PM	**0.51**	**0.70**	**0.36**	**0.50**	0.35	**0.73**	0.23	**0.39**	**0.44**	**0.67**
Mouse	Full	PM	0.50	0.68	**0.36**	0.49	**0.36**	0.72	**0.24**	0.38	**0.44**	**0.67**
Mouse	Mouse	T	**0.51**	0.68	**0.36**	0.48	0.35	0.71	0.23	0.37	0.42	0.64
Mouse	Full	T	0.49	0.67	**0.36**	0.48	**0.36**	0.71	**0.24**	0.37	0.42	0.64
Rat	Rat	PM	**0.44**	**0.73**	**0.28**	**0.48**	**0.26**	**0.77**	**0.16**	**0.35**	**0.49**	**0.58**
Rat	Full	PM	0.41	0.68	0.27	0.44	**0.26**	0.74	**0.16**	0.32	0.46	0.55
Rat	Rat	T	0.43	0.71	0.27	0.46	**0.26**	0.75	**0.16**	0.33	0.46	0.54
Rat	Full	T	0.40	0.68	0.27	0.44	**0.26**	0.73	**0.16**	0.31	0.45	0.54
Yeast	Yeast	PM	0.57	**0.81**	0.38	**0.59**	0.36	**0.86**	0.23	**0.44**	**0.60**	**0.68**
Yeast	Full	PM	**0.58**	**0.81**	**0.39**	**0.59**	**0.40**	**0.86**	**0.26**	0.42	**0.60**	**0.68**
Yeast	Yeast	T	0.54	0.79	0.37	0.56	0.36	0.84	0.23	0.39	0.55	0.64
Yeast	Full	T	0.55	0.78	0.38	0.57	0.38	0.83	0.25	0.39	0.55	0.65

[a] Human: 2,436 publications; Mouse: 2,309 publications; Rat: 1,796 publications; Yeast: 895 publications.

Tab. 21: Statistical significance (p-value) of the paired t-test between GOTA (PM, species-specific KB) and the other methods over BP

Species[a]	KB[b]	Info[c]	iP_1	iR_{10}	iP_{10}	hR_{10}	hP_{10}	MRR_{10}	R_{10}	$\#^e_{10}$
Human	Full	PM	**0.2581**	**0.5873**	**1**	**0.6833**	**1**	**0.09423**	**0.6476**	**0.9212**
Human	Human	T	0.001669	1.009e-11	1.938e-05	<2.2e-16	**0.4682**	6.024e-10	7.868e-12	5.668e-08
Human	Full	T	0.004912	0.0006756	**1**	0.003708	**1**	3.492e-06	0.001510	**0.06634**
Mouse	Full	PM	**0.06664**	5.23e-05	**0.9985**	0.0003303	**1**	0.02224	**0.1861**	**0.4773**
Mouse	Mouse	T	**0.2501**	8.74e-10	**0.7094**	1.652e-13	**0.7998**	3.543e-09	6.018e-10	8.489e-08
Mouse	Full	T	0.00329	2.822e-12	**0.8873**	1.440e-09	**1**	1.609e-06	0.0001651	0.0001967
Rat	Full	PM	2.841e-05	1.184e-11	0.005126	4.982e-10	**0.2569**	2.668e-06	3.764e-05	0.00179
Rat	Rat	T	**0.1173**	0.0001055	**0.2427**	5.467e-10	**0.882**	7.125e-08	5.181e-07	3.671e-07
Rat	Full	T	4.638e-07	6.224e-13	0.02333	8.001e-13	**0.219**	3.310e-09	1.699e-06	8.095e-05
Yeast	Full	PM	**0.7231**	**0.2576**	**1**	**0.1607**	**1**	0.008267	**0.4393**	**0.577**
Yeast	Yeast	T	1.49e-05	3.195e-05	0.01912	4.723e-05	**0.06464**	1.916e-15	5.768e-08	1.986e-05
Yeast	Full	T	0.009324	1.108e-05	**0.988**	5.47e-06	**1**	1.319e-09	3.728e-05	0.00231

Not significant p-values are highlighted in bold.

[a] Human: 2,436 publications; Mouse: 2,309 publications; Rat: 1,796 publications; Yeast: 895 publications.

Tab. 22: Performance comparison over MF

Species[a]	KB[b]	Info[c]	IT[d]			CAFA[d]	BC[d]			TREC[d]		$\#^e$
			iP_1	iR_{10}	iP_{10}	hF_{max}	hF_{10}	hR_{10}	hP_{10}	MRR_{10}	R_{10}	
Human	Human	PM	**0.63**	0.69	0.34	**0.69**	0.31	0.88	0.19	**0.63**	0.73	0.83
Human	Full	PM	0.62	**0.70**	**0.35**	0.68	**0.33**	**0.89**	**0.20**	0.61	**0.75**	**0.85**
Human	Human	T	0.59	0.67	0.34	0.68	0.30	0.85	0.18	0.60	0.69	0.79
Human	Full	T	0.62	0.69	0.34	0.68	0.31	0.88	0.19	0.62	0.73	0.83
Mouse	Mouse	PM	0.61	0.69	0.34	**0.69**	0.31	0.87	0.19	**0.60**	0.70	0.80
Mouse	Full	PM	**0.63**	**0.70**	**0.35**	0.68	**0.33**	**0.88**	**0.20**	**0.60**	**0.74**	**0.84**
Mouse	Mouse	T	0.57	0.69	0.34	0.67	0.31	0.86	0.19	0.57	0.68	0.79
Mouse	Full	T	0.61	0.69	0.34	0.68	**0.33**	**0.88**	**0.20**	**0.60**	0.73	0.83
Rat	Rat	PM	0.54	**0.73**	0.34	0.62	0.32	0.86	0.20	0.50	0.63	0.73
Rat	Full	PM	**0.56**	**0.73**	**0.35**	0.63	**0.35**	**0.87**	**0.22**	**0.51**	**0.66**	**0.77**
Rat	Rat	T	0.50	0.72	0.34	0.59	0.32	0.85	0.20	0.45	0.61	0.71
Rat	Full	T	0.55	0.72	**0.35**	**0.64**	0.34	0.86	0.21	**0.51**	**0.66**	**0.77**
Yeast	Yeast	PM	**0.58**	0.82	0.34	0.63	0.31	0.88	0.19	**0.46**	0.66	0.70
Yeast	Full	PM	**0.58**	**0.83**	**0.37**	**0.64**	**0.35**	**0.90**	**0.22**	0.45	**0.71**	**0.75**
Yeast	Yeast	T	0.56	0.80	0.34	0.60	0.31	0.87	0.19	0.41	0.62	0.67
Yeast	Full	T	0.53	0.82	0.36	0.60	0.34	0.88	0.21	0.39	0.67	0.71

[a] Human: 2,317 publications; Mouse: 1,291 publications; Rat: 858 publications; Yeast: 567 publications.

Tab. 23: Statistical significance (p-value) of the paired t-test between GOTA (PM, species-specific KB) and the other methods over MF

Species[a]	KB[b]	Info[c]	iP_1	iR_{10}	iP_{10}	hR_{10}	hP_{10}	MRR_{10}	R_{10}	$\#^e_{10}$
Human	Full	PM	**0.3633**	**0.7189**	**1**	**0.9587**	**1**	0.0005271	**0.9999**	**0.9998**
Human	Human	T	2.953e-15	1.940e-11	1.427e-08	<2.2e-16	0.02109	<2.2e-16	8.337e-15	1.656e-13
Human	Full	T	**0.3635**	0.03764	**0.8909**	**0.07189**	**0.9999**	0.01042	**0.7146**	**0.8425**
Mouse	Full	PM	**0.989**	**0.963**	**1**	**0.9943**	**1**	**0.6887**	**1**	**1**
Mouse	Mouse	T	3.257e-08	0.02194	0.01187	0.0004821	**0.07814**	1.252e-07	0.0001682	0.001578
Mouse	Full	T	**0.5398**	**0.5274**	**0.9935**	**0.8697**	**0.986**	**0.434**	**0.9962**	**0.9972**
Rat	Full	PM	**0.9475**	**0.6593**	**0.9984**	**0.9102**	**1**	**0.9569**	**0.9965**	**0.9997**
Rat	Rat	T	3.461e-06	0.02670	**0.08627**	0.01678	**0.869**	4.071e-09	0.0003287	0.009531
Rat	Full	T	**0.554**	**0.4772**	**0.9965**	**0.6382**	**1**	**0.8273**	**0.9919**	**0.9995**
Yeast	Full	PM	**0.3616**	**0.856**	**1**	**0.931**	**1**	**0.1598**	**0.9992**	**0.9985**
Yeast	Yeast	T	0.007898	0.005725	**0.2039**	0.00824	**0.0857**	2.515e-09	0.000652	0.001315
Yeast	Full	T	0.0001061	**0.5551**	**0.9989**	**0.4334**	**1**	3.779e-06	**0.7824**	**0.7275**

Not significant p-values are highlighted in bold.

[a] Human: ,2317 publications; Mouse: 1,291 publications; Rat: 858 publications; Yeast: 567 publications.

Tab. 24: Performance comparison over CC

Species[a]	KB[b]	Info[c]	IT[d]			CAFA[d]	BC[d]			TREC[d]		#[e]
			iP_1	iR_{10}	iP_{10}	hF_{max}	hF_{10}	hR_{10}	hP_{10}	MRR_{10}	R_{10}	
Human	Human	PM	**0.63**	**0.87**	**0.43**	**0.65**	**0.40**	**0.91**	**0.26**	**0.54**	**0.74**	**0.83**
Human	Full	PM	**0.63**	**0.87**	**0.43**	**0.65**	**0.40**	0.90	**0.26**	**0.54**	**0.74**	**0.83**
Human	Human	T	0.61	0.85	0.42	0.63	0.39	0.89	0.25	0.51	0.71	0.80
Human	Full	T	0.61	0.85	**0.43**	0.64	0.39	0.90	0.25	0.52	0.72	0.81
Mouse	Mouse	PM	**0.62**	**0.86**	0.41	**0.63**	**0.40**	**0.89**	**0.26**	**0.53**	**0.72**	**0.83**
Mouse	Full	PM	0.60	0.84	**0.42**	**0.63**	**0.40**	**0.89**	**0.26**	0.51	0.71	0.82
Mouse	Mouse	T	0.61	0.85	0.41	0.62	**0.40**	**0.89**	**0.26**	0.51	0.70	0.81
Mouse	Full	T	0.61	0.84	**0.42**	0.62	0.39	0.88	0.25	0.51	0.69	0.80
Rat	Rat	PM	0.60	0.83	**0.42**	0.61	0.39	0.87	0.25	0.47	0.66	0.78
Rat	Full	PM	**0.61**	**0.84**	**0.42**	**0.62**	**0.40**	**0.88**	**0.26**	**0.48**	**0.68**	**0.79**
Rat	Rat	T	0.60	0.83	**0.42**	0.61	**0.40**	0.86	**0.26**	0.47	0.64	0.76
Rat	Full	T	0.60	0.83	**0.42**	0.61	0.39	0.87	0.25	**0.48**	0.65	0.77
Yeast	Yeast	PM	**0.65**	**0.88**	**0.46**	0.68	0.42	**0.93**	0.27	**0.54**	**0.76**	0.81
Yeast	Full	PM	0.64	**0.88**	**0.46**	**0.69**	**0.43**	0.92	**0.28**	0.53	**0.76**	**0.82**
Yeast	Yeast	T	0.63	**0.88**	0.45	0.67	0.41	0.92	0.26	0.52	0.73	0.79
Yeast	Full	T	0.63	0.87	**0.46**	0.67	**0.43**	0.92	**0.28**	0.51	0.74	0.80

[a] Human: 1,426 publications; Mouse: 1,148 publications; Rat: 702 publications; Yeast: 517 publications.

Tab. 25: Statistical significance (p-value) of the paired t-test between GOTA (PM, species-specific KB) and the other methods over CC

Species[a]	KB[b]	Info[c]	iP_1	iR_{10}	iP_{10}	hR_{10}	hP_{10}	MRR_{10}	R_{10}	$\#^e_{10}$
Human	Full	PM	**0.4694**	**0.1387**	**0.8534**	**0.1269**	**0.7785**	**0.6434**	**0.5947**	**0.5718**
Human	Human	T	2.734e-05	2.105e-08	3.819e-09	7.425e-10	0.0002031	2.421e-09	9.9e-07	1.009e-05
Human	Full	T	0.002206	9.299e-05	0.01403	0.001152	**0.2820**	0.001040	0.00731	0.01627
Mouse	Full	PM	0.009212	0.005181	**0.9999**	**0.1063**	9.696e-06	0.03861	**0.1657**	**0.1922**
Mouse	Mouse	T	0.005044	0.04675	**0.2095**	0.009322	**0.3462**	0.0002413	0.03106	0.02836
Mouse	Full	T	0.02036	5.513e-05	**0.9637**	0.008063	1.612e-08	0.01039	0.008244	0.003736
Rat	Full	PM	**0.8373**	**0.8174**	**0.7145**	**0.8948**	**0.9139**	**0.8484**	**0.918**	**0.8173**
Rat	Rat	T	**0.552**	0.02819	**0.5942**	0.04796	**0.8859**	**0.1345**	0.02786	0.01736
Rat	Full	T	**0.6496**	**0.08866**	**0.1658**	**0.2743**	**0.4234**	**0.6788**	**0.2880**	**0.1406**
Yeast	Full	PM	**0.4217**	**0.2377**	**0.8465**	**0.1431**	**1**	**0.1868**	**0.4292**	**0.6158**
Yeast	Yeast	T	0.03855	0.04735	0.001149	**0.05504**	**0.06051**	0.0008659	0.003346	0.03591
Yeast	Full	T	0.03639	0.0385	**0.2468**	0.01245	**1**	0.007506	**0.06752**	**0.2504**

Not significant p-values are highlighted in bold.

[a] Human: 1,426 publications; Mouse: 1,148 publications; Rat: 702 publications; Yeast: 517 publications.

[b] Knowledge base used for prediction. Full = all available publications in the KB. Human/Mouse/Rat/Yeast = only publications related to Human/Mouse/Rat/Yeast.

[c] Informations used in prediction: PM = title, abstract, references and publication year (PubMed); T = title.

[d] Metrics definitions are in Section 2.3. For each metric and Species, the best result is highlighted in bold.

[e] Fraction of publications for which at least one gold-standard annotation has been correctly predicted.

2.2.2 Evaluation of the top-20 predicted GO terms

Tab. 26: Performance comparison over all GO

Species[a]	KB[b]	Info[c]	IT[d]			CAFA[d]	BC[d]			TREC[d]		#[e]
			iP_1	iR_{20}	iP_{20}	hF_{max}	hF_{20}	hR_{20}	hP_{20}	MRR_{20}	R_{20}	
Human	Human	PM	**0.45**	**0.69**	**0.25**	**0.46**	0.27	**0.79**	0.16	**0.49**	**0.59**	0.82
Human	Full	PM	0.44	**0.69**	**0.25**	0.44	**0.28**	**0.79**	**0.17**	0.45	**0.59**	**0.83**
Human	Human	T	0.42	0.67	0.24	0.45	0.26	0.76	0.16	0.46	0.55	0.79
Human	Full	T	0.44	**0.69**	**0.25**	0.44	**0.28**	0.78	**0.17**	0.45	0.57	0.81
Mouse	Mouse	PM	**0.45**	**0.72**	**0.27**	**0.45**	**0.29**	**0.77**	**0.18**	**0.46**	**0.53**	**0.82**
Mouse	Full	PM	**0.45**	0.70	**0.27**	0.44	**0.29**	**0.77**	**0.18**	0.44	**0.53**	0.81
Mouse	Mouse	T	0.42	0.70	0.26	0.44	**0.29**	0.76	**0.18**	0.43	0.51	0.80
Mouse	Full	T	0.44	0.69	**0.27**	0.43	**0.29**	0.76	**0.18**	0.43	0.51	0.80
Rat	Rat	PM	**0.38**	**0.72**	0.19	**0.41**	0.21	**0.79**	0.12	**0.36**	**0.53**	**0.69**
Rat	Full	PM	0.34	0.70	0.19	0.37	0.21	0.77	0.12	0.33	0.51	0.67
Rat	Rat	T	0.37	0.71	**0.20**	0.40	**0.22**	0.77	**0.13**	0.34	0.51	0.67
Rat	Full	T	0.33	0.70	0.19	0.37	0.21	0.77	0.12	0.33	0.51	0.66
Yeast	Yeast	PM	**0.45**	**0.80**	0.25	**0.47**	0.27	**0.85**	0.16	**0.42**	**0.61**	**0.76**
Yeast	Full	PM	0.43	0.79	**0.26**	**0.47**	**0.30**	**0.85**	**0.18**	0.40	**0.61**	**0.76**
Yeast	Yeast	T	0.41	0.78	0.24	0.44	0.27	0.83	0.16	0.37	0.56	0.71
Yeast	Full	T	0.41	0.78	0.25	0.44	0.28	0.84	0.17	0.36	0.58	0.73

[a] Human: 3,575 publications; Mouse: 2,825 publications; Rat: 2,380 publications; Yeast: 1,290 publications.

Tab. 27: Statistical significance (p-value) of the paired t-test between GOTA (PM,species-specific KB) and the other methods over all GO

Species[a]	KB[b]	Info[c]	iP_1	iR_{20}	iP_{20}	hR_{20}	hP_{20}	MRR_{20}	R_{20}	$\#^e_{20}$
Human	Full	PM	0.02073	**0.5773**	**1**	**0.7016**	**1**	<2.2e-16	**0.3049**	**0.8882**
Human	Human	T	1.659e-12	<2.2e-16	2.611e-08	<2.2e-16	0.004045	<2.2e-16	<2.2e-16	5.57e-14
Human	Full	T	0.008581	0.00807	**1**	0.0001703	**0.999**	<2.2e-16	8.443e-08	0.008668
Mouse	Full	PM	**0.568**	4.597e-05	**1**	**0.05988**	**1**	8.839e-06	**0.3077**	**0.1478**
Mouse	Mouse	T	3.392e-07	9.704e-08	0.02077	1.68e-14	**0.5211**	8.716e-10	9.857e-10	2.914e-06
Mouse	Full	T	0.02507	6.501e-12	**0.8236**	9.279e-08	**1**	5.375e-09	2.827e-05	0.0001964
Rat	Full	PM	2.692e-09	4.657e-08	**0.092**	8.969e-06	**0.1533**	8.35e-08	0.001245	0.01018
Rat	Rat	T	0.04483	0.0002362	**0.9483**	1.243e-07	**0.9806**	4.020e-08	4.435e-06	2.913e-05
Rat	Full	T	1.285e-10	4.446e-07	0.03418	1.144e-06	**0.3201**	1.661e-08	7.542e-05	0.001934
Yeast	Full	PM	0.00985	**0.2729**	**1**	**0.3504**	**1**	0.0001778	**0.6285**	**0.5676**
Yeast	Yeast	T	4.792e-07	6.787e-08	0.0009326	5.423e-07	0.001745	<2.2e-16	2.459e-14	2.375e-10
Yeast	Full	T	8.055e-07	0.0004433	**0.9957**	0.0008086	**1**	4.944e-16	0.0006018	0.003941

Not significant p-values are highlighted in bold

[a] Human: 3,575 publications; Mouse: 2,825 publications; Rat: 2,380 publications; Yeast: 1,290 publications.

Tab. 28: Performance comparison over BP

Species[a]	KB[b]	Info[c]	IT[d]			CAFA[d]	BC[d]			TREC[d]		#[e]
			iP_1	iR_{20}	iP_{20}	hF_{max}	hF_{20}	hR_{20}	hP_{20}	MRR_{20}	R_{20}	
Human	Human	PM	**0.49**	**0.76**	**0.30**	**0.48**	0.25	0.82	0.15	**0.36**	0.54	0.70
Human	Full	PM	0.48	**0.76**	**0.30**	**0.48**	**0.27**	**0.83**	**0.16**	**0.36**	**0.55**	**0.71**
Human	Human	T	0.47	0.74	0.29	0.47	0.25	0.79	0.15	0.34	0.50	0.66
Human	Full	T	0.47	0.75	**0.30**	0.47	**0.27**	0.82	**0.16**	0.34	0.52	0.68
Mouse	Mouse	PM	**0.51**	**0.76**	**0.31**	**0.50**	0.25	**0.81**	0.15	**0.40**	0.52	0.73
Mouse	Full	PM	0.50	**0.76**	**0.31**	0.49	**0.27**	**0.81**	**0.16**	0.39	**0.54**	**0.75**
Mouse	Mouse	T	**0.51**	0.75	**0.31**	0.48	**0.27**	0.80	**0.16**	0.37	0.50	0.72
Mouse	Full	T	0.49	0.75	**0.31**	0.48	**0.27**	0.80	**0.16**	0.37	0.51	0.73
Rat	Rat	PM	**0.44**	**0.78**	**0.24**	**0.48**	**0.18**	**0.84**	**0.10**	**0.35**	**0.56**	**0.65**
Rat	Full	PM	0.41	0.75	0.23	0.44	**0.18**	0.82	**0.10**	0.32	0.54	0.63
Rat	Rat	T	0.43	**0.78**	**0.24**	0.46	**0.18**	0.83	**0.10**	0.33	0.54	0.62
Rat	Full	T	0.40	0.75	0.23	0.44	**0.18**	0.81	**0.10**	0.31	0.52	0.61
Yeast	Yeast	PM	0.57	**0.86**	0.33	**0.59**	0.26	**0.92**	0.15	**0.45**	0.67	0.76
Yeast	Full	PM	**0.58**	**0.86**	**0.34**	**0.59**	**0.29**	**0.92**	**0.17**	0.43	**0.69**	**0.77**
Yeast	Yeast	T	0.54	0.84	0.32	0.56	0.26	0.90	0.15	0.39	0.63	0.71
Yeast	Full	T	0.55	0.84	0.33	0.57	**0.29**	0.90	**0.17**	0.39	0.65	0.73

[a] Human: 2,436 publications; Mouse: 2,309 publications; Rat: 1,796 publications; Yeast: 895 publications.

Tab. 29: Statistical significance (p-value) of the paired t-test between GOTA (PM,species-specific KB) and the other methods over BP

Species[a]	KB[b]	Info[c]	iP_1	iR_{20}	iP_{20}	hR_{20}	hP_{20}	MRR_{20}	R_{20}	$\#^e_{20}$
Human	Full	PM	**0.2581**	**0.6517**	**1**	**0.8138**	**1**	**0.1012**	**0.9542**	**0.9618**
Human	Human	T	0.001669	<2.2e-16	5.076e-06	<2.2e-16	**0.4166**	2.771e-10	<2.2e-16	2.072e-11
Human	Full	T	0.004912	0.002397	**1**	0.006948	**1**	2.497e-06	0.006966	0.0481
Mouse	Full	PM	**0.06664**	**0.3218**	**1**	**0.6621**	**1**	0.03738	**0.9992**	**0.9959**
Mouse	Mouse	T	**0.2501**	2.355e-05	**0.995**	3.11e-07	**1**	8.52e-09	8.686e-08	0.0005025
Mouse	Full	T	0.00329	0.0008142	**1**	0.00588	**1**	4.668e-06	**0.1925**	**0.3413**
Rat	Full	PM	2.841e-05	2.526e-10	0.007573	1.978e-06	**0.994**	2.557e-06	0.004912	0.01336
Rat	Rat	T	**0.1173**	0.009229	**0.7354**	1.250e-05	**0.9994**	8.657e-08	0.001126	9.98e-05
Rat	Full	T	4.638e-07	3.403e-12	0.008482	5.066e-11	**0.963**	2.472e-09	5.48e-05	0.0001880
Yeast	Full	PM	**0.7231**	**0.5231**	**1**	**0.4641**	**1**	0.009858	**0.964**	**0.8594**
Yeast	Yeast	T	1.49e-05	5.794e-05	**0.08784**	2.329e-05	**0.1619**	6.818e-16	2.175e-08	3.403e-08
Yeast	Full	T	0.009324	9.836e-05	**0.9997**	0.001809	**1**	1.154e-09	0.01908	0.009246

Not significant p-values are highlighted in bold.

[a] Human: 2,436 publications; Mouse: 2,309 publications; Rat: 1,796 publications; Yeast: 895 publications.

Tab. 30: Performance comparison over MF

Species[a]	KB[b]	Info[c]	IT[d]			CAFA[d]	BC[d]			TREC[d]		$\#^e$
			iP_1	iR_{20}	iP_{20}	hF_{max}	hF_{20}	hR_{20}	hP_{20}	MRR_{20}	R_{20}	
Human	Human	PM	**0.63**	**0.73**	0.30	**0.69**	**0.20**	0.92	**0.11**	**0.63**	0.78	0.86
Human	Full	PM	0.62	**0.73**	**0.31**	0.68	**0.20**	**0.93**	**0.11**	0.61	**0.81**	**0.88**
Human	Human	T	0.59	0.71	0.30	0.68	**0.20**	0.90	**0.11**	0.60	0.75	0.84
Human	Full	T	0.62	0.72	0.30	0.68	**0.20**	0.92	**0.11**	0.62	0.79	0.87
Mouse	Mouse	PM	0.61	0.73	0.30	**0.69**	0.20	0.91	0.11	0.60	0.75	0.84
Mouse	Full	PM	**0.63**	**0.74**	**0.31**	0.68	**0.21**	**0.92**	**0.12**	**0.61**	**0.80**	**0.88**
Mouse	Mouse	T	0.57	0.72	0.29	0.67	0.20	0.90	0.11	0.58	0.73	0.82
Mouse	Full	T	0.61	0.73	0.30	0.68	0.20	**0.92**	0.11	0.60	0.78	0.86
Rat	Rat	PM	0.54	**0.77**	0.30	0.62	0.21	**0.91**	0.12	0.50	0.69	0.77
Rat	Full	PM	**0.56**	**0.77**	**0.31**	0.63	**0.23**	**0.91**	**0.13**	**0.52**	**0.74**	**0.83**
Rat	Rat	T	0.50	0.76	0.29	0.59	0.21	0.90	0.12	0.46	0.67	0.76
Rat	Full	T	0.55	**0.77**	0.30	**0.64**	**0.23**	0.90	**0.13**	0.51	**0.74**	**0.83**
Yeast	Yeast	PM	**0.58**	0.84	0.30	0.63	0.20	0.92	0.11	**0.46**	0.71	0.75
Yeast	Full	PM	**0.58**	**0.86**	**0.32**	**0.64**	**0.23**	**0.93**	**0.13**	0.45	**0.78**	**0.81**
Yeast	Yeast	T	0.56	0.84	0.30	0.60	0.20	0.91	0.11	0.41	0.70	0.74
Yeast	Full	T	0.53	0.85	0.31	0.60	0.21	0.92	0.12	0.40	0.75	0.78

[a] Human: 2,317 publications; Mouse: 1,291 publications; Rat: 858 publications; Yeast: 567 publications.

Tab. 31: Statistical significance (p-value) of the paired t-test between GOTA (PM, species-specific KB) and the other methods over MF

Species[a]	KB[b]	Info[c]	iP_1	iR_{20}	iP_{20}	hR_{20}	hP_{20}	MRR_{20}	R_{20}	$\#^e_{20}$
Human	Full	PM	**0.3633**	**0.08656**	**0.9944**	**0.9522**	**1**	0.0005079	**1**	**1**
Human	Human	T	2.953e-15	5.72e-12	1.836e-11	5.663e-15	0.03279	<2.2e-16	7.803e-13	9.65e-10
Human	Full	T	**0.3635**	3.096e-05	**0.05813**	0.04899	**0.9656**	0.01180	**0.8784**	**0.9728**
Mouse	Full	PM	**0.989**	**0.9931**	**1**	**0.9996**	**1**	**0.7002**	**1**	**1**
Mouse	Mouse	T	3.257e-08	0.01144	0.0002722	0.001962	**0.1830**	8.506e-08	4.678e-07	0.0001032
Mouse	Full	T	**0.5398**	**0.7493**	**0.9893**	**0.9358**	**1**	**0.4327**	**0.9986**	**0.9971**
Rat	Full	PM	**0.9475**	**0.5231**	**1**	**0.7157**	**1**	**0.965**	**1**	**1**
Rat	Rat	T	3.461e-06	0.0008576	0.001456	0.0008643	**0.7952**	2.948e-09	0.0003527	0.009772
Rat	Full	T	**0.554**	**0.3089**	**0.9963**	**0.3789**	**1**	**0.8453**	**0.9998**	**1**
Yeast	Full	PM	**0.3616**	**0.9973**	**1**	**0.9966**	**1**	**0.1692**	**1**	**1**
Yeast	Yeast	T	0.007898	**0.4083**	**0.581**	**0.09805**	**0.4584**	3.609e-09	0.01883	0.04163
Yeast	Full	T	0.0001061	**0.9227**	**1**	**0.906**	**1**	4.909e-06	**0.9657**	**0.967**

Not significant p-values are highlighted in bold.

[a] Human: 2,317 publications; Mouse: 1,291 publications; Rat: 858 publications; Yeast: 567 publications.

Tab. 32: Performance comparison over CC

Species[a]	KB[b]	Info[c]	IT[d]			CAFA[d]	BC[d]			TREC[d]		#[e]
			iP_1	iR_{20}	iP_{20}	hF_{max}	hF_{20}	hR_{20}	hP_{20}	MRR_{20}	R_{20}	
Human	Human	PM	**0.63**	**0.91**	**0.38**	**0.65**	**0.27**	**0.95**	**0.16**	0.54	0.82	**0.89**
Human	Full	PM	**0.63**	**0.91**	**0.38**	**0.65**	**0.27**	**0.95**	**0.16**	**0.55**	**0.83**	**0.89**
Human	Human	T	0.61	0.89	0.37	0.63	0.26	0.93	0.15	0.51	0.79	0.86
Human	Full	T	0.61	0.90	0.37	0.64	**0.27**	0.94	**0.16**	0.52	0.80	0.87
Mouse	Mouse	PM	**0.62**	**0.91**	**0.37**	**0.63**	**0.27**	**0.94**	**0.16**	**0.53**	**0.81**	**0.89**
Mouse	Full	PM	0.60	0.89	**0.37**	**0.63**	**0.27**	**0.94**	**0.16**	0.52	0.80	**0.89**
Mouse	Mouse	T	0.61	0.90	0.36	0.62	**0.27**	**0.94**	**0.16**	0.52	0.79	0.88
Mouse	Full	T	0.61	0.88	**0.37**	0.62	**0.27**	0.93	**0.16**	0.52	0.79	0.88
Rat	Rat	PM	0.60	0.87	**0.38**	0.61	**0.27**	0.92	**0.16**	0.48	0.75	0.85
Rat	Full	PM	**0.61**	**0.88**	**0.38**	**0.62**	**0.27**	**0.93**	**0.16**	**0.49**	**0.77**	**0.87**
Rat	Rat	T	0.60	0.87	**0.38**	0.61	**0.27**	0.92	**0.16**	0.47	0.74	0.85
Rat	Full	T	0.60	**0.88**	**0.38**	0.61	**0.27**	0.92	**0.16**	0.48	**0.77**	0.86
Yeast	Yeast	PM	**0.65**	**0.92**	**0.41**	0.68	0.29	**0.96**	0.17	**0.55**	**0.83**	**0.87**
Yeast	Full	PM	0.64	**0.92**	**0.41**	**0.69**	**0.30**	**0.96**	**0.18**	0.54	**0.83**	**0.87**
Yeast	Yeast	T	0.63	0.91	0.40	0.67	0.29	0.95	0.17	0.52	0.81	0.85
Yeast	Full	T	0.63	0.91	**0.41**	0.67	**0.30**	0.95	**0.18**	0.52	0.82	**0.87**

[a] Human: 1,426 publications; Mouse: 1,148 publications; Rat: 702 publications; Yeast: 517 publications.

Tab. 33: Statistical significance (p-value) of the paired t-test between GOTA (species-specific KB) and the other methods over CC

Species[a]	KB[b]	Info[c]	iP_1	iR_{20}	iP_{20}	hR_{20}	hP_{20}	MRR_{20}	R_{20}	$\#^e_{20}$
Human	Full	PM	**0.4694**	**0.09161**	**0.7452**	**0.2115**	**0.9875**	**0.6511**	**0.92**	**0.7684**
Human	Human	T	2.734e-05	1.072e-10	2.256e-14	1.221e-09	2.38e-10	1.558e-09	1.51e-10	3.719e-09
Human	Full	T	0.002206	0.0002005	0.0002129	0.005731	**0.08008**	0.001007	0.01254	0.01547
Mouse	Full	PM	0.009212	0.004475	**1**	0.01225	**0.2568**	0.03845	**0.2375**	**0.3095**
Mouse	Mouse	T	0.005044	0.0001503	5.79e-05	7.565e-05	0.004472	0.0002427	0.001004	0.02774
Mouse	Full	T	0.02036	1.240e-05	**0.9845**	0.000637	0.002810	0.01199	0.00908	0.03592
Rat	Full	PM	**0.8373**	**0.9476**	**0.4139**	**0.837**	**0.9601**	**0.8579**	**0.9856**	**0.9015**
Rat	Rat	T	**0.552**	**0.376**	**0.5671**	**0.1759**	**0.839**	**0.1715**	**0.2235**	**0.1378**
Rat	Full	T	**0.6496**	**0.8832**	**0.4821**	**0.5842**	**0.7023**	**0.7307**	**0.9265**	**0.6913**
Yeast	Full	PM	**0.4217**	**0.2693**	**0.9995**	**0.2292**	**1**	**0.1746**	**0.4142**	**0.373**
Yeast	Yeast	T	0.03855	0.001203	0.03608	0.01495	**0.1372**	0.0007548	0.03622	0.03025
Yeast	Full	T	0.03639	0.04077	**0.7963**	0.04279	**1**	0.00732	**0.2324**	**0.3311**

Not significant p-values are highlighted in bold.

[a] Human: 1426 publications; Mouse: 1148 publications; Rat: 702 publications; Yeast: 517 publications.

[b] Knowledge base used for prediction. Full = all available publications in the KB. Human/Mouse/Rat/Yeast = only publications related to Human/Mouse/Rat/Yeast.

[c] Informations used in prediction: PM = title, abstract, references and publication year (PubMed); T = title.

[d] Metrics definitions are in Section 2.3. For each metric and Species, the best result is highlighted in bold.

[e] Fraction of publications for which at least one gold-standard annotation has been correctly predicted.

2.3 Performance comparison with related approaches

2.3.1 Evaluation of the top-10 predicted GO terms

Tab. 34: Performance comparison over all GO (412 publications)

Method[a]	Info[b]	IT[c]			CAFA[c]	BC[c]			TREC[c]		$\#_{10}^{d}$
		iP_1	iR_{10}	iP_{10}	hF_{max}	hF_{10}	hR_{10}	hP_{10}	MRR_{10}	R_{10}	
GOTA	PM	**0.42**	**0.69**	**0.26**	**0.42**	**0.31**	**0.73**	**0.20**	**0.39**	**0.49**	**0.62**
GOTA	T+A	0.37	0.68	**0.26**	0.41	**0.31**	**0.73**	**0.20**	0.35	0.48	**0.62**
GOTA	T	0.39	0.66	0.24	0.39	0.30	0.70	0.19	0.34	0.44	0.58
GOCat	T+A	0.34	0.64	0.23	0.37	0.30	0.69	0.19	0.29	0.40	0.52
GOCat	T	0.30	0.64	0.22	0.36	0.30	0.69	0.19	0.28	0.40	0.53
RandFR	N/A	0.08	0.21	0.08	0.10	0.10	0.23	0.06	0.03	0.05	0.08
RandIC	N/A	0.22	0.23	0.17	0.19	0.15	0.30	0.10	0.00	0.01	0.01

Tab. 35: Statistical significance (p-value) of the paired t-test between GOTA (PM) and the other methods over all GO (412 publications)

Method[a]	Info[b]	iP_1	iR_{10}	iP_{10}	hR_{10}	hP_{10}	MRR_{10}	R_{10}	$\#_{10}$
GOTA	T+A	8.399e-06	**0.0884**	0.009854	**0.1800**	**0.4151**	1.895e-06	**0.06748**	**0.3011**
GOTA	T	0.005682	0.0003516	1.433e-08	0.0005758	0.002742	4.823e-06	0.0002105	0.002672
GOCat	T+A	0.0001621	0.0006705	8.252e-09	0.001465	**0.05882**	5.337e-07	1.002e-05	6.75e-05
GOCat	T	1.532e-08	0.0002619	1.772e-15	0.0008314	0.04761	1.502e-10	1.377e-06	1.868e-05
RandFR	N/A	<2.2e-16	<2.2e-16	<2.2e-16	<2.2e-16	<2.2e-16	<2.2e-16	<2.2e-16	<2.2e-16
RandIC	N/A	<2.2e-16	<2.2e-16	<2.2e-16	<2.2e-16	<2.2e-16	<2.2e-16	<2.2e-16	<2.2e-16

Not significant p-values are highlighted in bold.

Tab. 36: Performance comparison over BP (323 publications)

Method[a]	Info[b]	IT[c]			CAFA[c]	BC[c]			TREC[c]		$\#_{10}^{d}$
		iP_1	iR_{10}	iP_{10}	hF_{max}	hF_{10}	hR_{10}	hP_{10}	MRR_{10}	R_{10}	
GOTA	PM	**0.52**	**0.76**	**0.34**	**0.52**	**0.32**	**0.81**	**0.20**	**0.41**	**0.56**	**0.65**
GOTA	T+A	0.49	0.75	**0.34**	**0.52**	**0.32**	0.80	**0.20**	0.38	0.54	0.63
GOTA	T	0.48	0.72	0.33	0.49	0.31	0.78	0.19	0.37	0.50	0.60
GOCat	T+A	0.49	0.72	0.33	0.49	0.30	0.77	0.19	0.34	0.47	0.57
GOCat	T	0.50	0.69	0.31	0.49	0.29	0.75	0.18	0.33	0.44	0.54
RandFR	N/A	0.14	0.30	0.15	0.13	0.11	0.31	0.07	0.01	0.03	0.04
RandIC	N/A	0.28	0.30	0.24	0.26	0.18	0.38	0.12	0.00	0.00	0.00

Tab. 37: Statistical significance (p-value) of the paired t-test between GOTA (PM) and the other methods over BP (323 publications)

Method[a]	Info[b]	iP_1	iR_{10}	iP_{10}	hR_{10}	hP_{10}	MRR_{10}	R_{10}	$\#_{10}$
GOTA	T+A	0.01154	**0.1967**	**0.3935**	**0.1522**	**0.6105**	0.0007866	**0.06437**	**0.137**
GOTA	T	0.003125	0.000684	0.006526	0.002783	0.002141	0.0001912	0.0002183	0.001476
GOCat	T+A	**0.1187**	0.005677	**0.1041**	0.002895	0.008732	0.0002507	8.948e-05	0.0008239
GOCat	T	**0.2011**	8.96e-06	2.575e-05	2.727e-06	5.808e-07	1.185e-05	3.019e-07	1.328e-05
RandFR	N/A	<2.2e-16	<2.2e-16	<2.2e-16	<2.2e-16	<2.2e-16	<2.2e-16	<2.2e-16	<2.2e-16
RandIC	N/A	<2.2e-16	<2.2e-16	<2.2e-16	<2.2e-16	<2.2e-16	<2.2e-16	<2.2e-16	<2.2e-16

Not significant p-values are highlighted in bold.

Tab. 38: Performance comparison over MF (138 publications)

Method[a]	Info[b]	IT[c]			CAFA[c]	BC[c]			TREC[c]		$\#_{10}^{d}$
		iP_1	iR_{10}	iP_{10}	hF_{max}	hF_{10}	hR_{10}	hP_{10}	MRR_{10}	R_{10}	
GOTA	PM	**0.68**	**0.86**	**0.38**	**0.71**	**0.37**	**0.90**	0.23	**0.59**	**0.75**	**0.80**
GOTA	T+A	0.59	0.85	0.37	0.67	**0.37**	**0.90**	0.23	0.51	0.74	0.78
GOTA	T	0.58	0.85	0.37	0.66	0.35	0.89	0.22	0.51	0.72	0.76
GOCat	T+A	0.54	0.81	0.33	0.63	0.32	0.86	0.20	0.42	0.62	0.64
GOCat	T	0.51	0.79	0.33	0.62	0.32	0.85	0.20	0.41	0.62	0.65
RandFR	N/A	0.25	0.41	0.23	0.31	0.16	0.47	0.10	0.06	0.12	0.14
RandIC	N/A	0.25	0.24	0.25	0.31	0.31	0.23	**0.48**	0.04	0.03	0.04

Tab. 39: Statistical significance (p-value) of the paired t-test between GOTA (PM) and the other methods over MF (138 publications)

Method[a]	Info[b]	iP_1	iR_{10}	iP_{10}	hR_{10}	hP_{10}	MRR_{10}	R_{10}	$\#_{10}$
GOTA	T+A	0.0001881	**0.2590**	0.04758	**0.2639**	**0.6661**	1.400e-05	**0.1951**	**0.2081**
GOTA	T	0.0001762	**0.1346**	**0.08272**	**0.07099**	**0.1507**	8.784e-06	0.04417	0.02925
GOCat	T+A	8.935e-05	0.01532	2.177e-06	0.02259	0.0008875	1.146e-06	0.0002914	5.817e-05
GOCat	T	1.639e-06	0.001997	6.314e-08	0.00398	0.004182	1.832e-07	0.0001350	9.793e-05
RandFR	N/A	<2.2e-16	<2.2e-16	<2.2e-16	<2.2e-16	<2.2e-16	<2.2e-16	<2.2e-16	<2.2e-16
RandIC	N/A	<2.2e-16	<2.2e-16	3.231e-10	<2.2e-16	**1**	<2.2e-16	<2.2e-16	<2.2e-16

Not significant p-values are highlighted in bold.

Tab. 40: Performance comparison over CC (83 publications)

Method[a]	Info[b]	IT[c]			CAFA[c]	BC[c]			TREC[c]		$\#_{10}^{d}$
		iP_1	iR_{10}	iP_{10}	hF_{max}	hF_{10}	hR_{10}	hP_{10}	MRR_{10}	R_{10}	
GOTA	PM	**0.58**	**0.86**	**0.43**	**0.64**	0.44	**0.90**	0.29	**0.44**	**0.68**	**0.71**
GOTA	T+A	0.56	0.85	0.42	0.62	0.43	0.89	0.28	0.43	0.67	0.70
GOTA	T	0.52	0.83	0.41	0.59	0.42	0.88	0.28	0.37	0.62	0.65
GOCat	T+A	0.50	0.81	0.39	0.60	0.40	0.86	0.26	0.37	0.62	0.66
GOCat	T	0.50	0.78	0.38	0.58	0.40	0.84	0.26	0.34	0.56	0.60
RandFR	N/A	0.41	0.62	0.33	0.47	0.34	0.66	0.23	0.16	0.37	0.40
RandIC	N/A	0.42	0.41	0.42	0.49	**0.49**	0.47	**0.51**	0.01	0.01	0.01

Tab. 41: Statistical significance (p-value) of the paired t-test between GOTA (PM) and the other methods over CC (83 publications)

Method[a]	Info[b]	iP_1	iR_{10}	iP_{10}	hR_{10}	hP_{10}	MRR_{10}	R_{10}	$\#_{10}$
GOTA	T+A	**0.2288**	**0.1858**	**0.08497**	**0.1424**	**0.4003**	**0.2762**	**0.2351**	**0.3287**
GOTA	T	0.0281	0.03741	0.03818	0.02834	**0.3452**	0.01465	**0.06625**	**0.06625**
GOCat	T+A	0.02166	0.04604	0.004627	**0.05275**	0.03565	**0.06891**	**0.1147**	**0.1985**
GOCat	T	0.02317	0.00455	0.0002335	0.01222	0.01942	0.01131	0.01315	0.02446
RandFR	N/A	1.049e-05	1.887e-09	1.179e-10	1.390e-10	0.0003039	4.869e-08	3.026e-06	4.621e-06
RandIC	N/A	3.247e-05	<2.2e-16	**0.3656**	<2.2e-16	**1**	2.337e-14	<2.2e-16	<2.2e-16

Not significant p-values are highlighted in bold.

[a] Method used for prediction. RandFR and RandIC are baseline predictors.

[b] Informations used in prediction: PM = title, abstract, references and publication year (PubMed); T+A = title and abstract; T = title; N/A = no information.

[c] Metrics definitions are in Section 2.3. For each metric, the best result is highlighted in bold.

[d] Fraction of publications for which at least one gold-standard annotation has been correctly predicted.

2.3.2 Evaluation of the top-20 predicted GO terms

Tab. 42: Performance comparison over all GO (412 publications)

Method[a]	Info[b]	IT[c]			CAFA[c]	BC[c]			TREC[c]		$\#_{20}^{d}$
		iP_1	iR_{20}	iP_{20}	hF_{max}	hF_{20}	hR_{20}	hP_{20}	MRR_{20}	R_{20}	
GOTA	PM	**0.42**	**0.79**	**0.23**	**0.42**	**0.24**	**0.83**	**0.14**	**0.39**	**0.61**	**0.73**
GOTA	T+A	0.37	0.77	0.22	0.41	**0.24**	**0.83**	**0.14**	0.35	0.58	0.71
GOTA	T	0.39	0.75	0.21	0.39	0.22	0.80	0.13	0.35	0.55	0.67
GOCat	T+A	0.34	0.74	0.20	0.37	0.22	0.79	0.13	0.30	0.52	0.65
GOCat	T	0.30	0.71	0.19	0.36	0.21	0.77	0.12	0.28	0.48	0.60
RandFR	N/A	0.08	0.32	0.08	0.10	0.10	0.33	0.06	0.03	0.07	0.11
RandIC	N/A	0.22	0.27	0.17	0.19	0.09	0.36	0.05	0.00	0.01	0.01

Tab. 43: Statistical significance (p-value) of the paired t-test between GOTA (PM) and the other methods over all GO (412 publications)

Method[a]	Info[b]	iP_1	iR_{20}	iP_{20}	hR_{20}	hP_{20}	MRR_{20}	R_{20}	$\#_{20}^{d}$
GOTA	T+A	8.399e-06	0.01669	0.02706	**0.1190**	**0.5**	9.196e-07	0.02083	**0.1006**
GOTA	T	0.005682	0.0001389	1.387e-08	0.0009283	0.0002931	3.388e-06	1.467e-05	0.0006481
GOCat	T+A	0.0001621	0.000708	1.985e-08	0.001911	0.01285	4.999e-07	3.811e-05	0.0006538
GOCat	T	1.532e-08	1.595e-08	4.424e-16	2.823e-07	1.473e-05	4.546e-11	8.111e-10	8.301e-08
RandFR	N/A	<2.2e-16	<2.2e-16	<2.2e-16	<2.2e-16	<2.2e-16	<2.2e-16	<2.2e-16	<2.2e-16
RandIC	N/A	<2.2e-16	<2.2e-16	1.532e-14	<2.2e-16	<2.2e-16	<2.2e-16	<2.2e-16	<2.2e-16

Not significant p-values are highlighted in bold.

Tab. 44: Performance comparison over BP (323 publications)

Method[a]	Info[b]	IT[c]			CAFA[c]	BC[c]			TREC[c]		$\#_{20}^{d}$
		iP_1	iR_{20}	iP_{20}	hF_{max}	hF_{20}	hR_{20}	hP_{20}	MRR_{20}	R_{20}	
GOTA	PM	**0.52**	**0.81**	**0.30**	**0.52**	**0.23**	**0.87**	**0.13**	**0.42**	**0.64**	**0.73**
GOTA	T+A	0.49	0.80	0.29	**0.52**	**0.23**	**0.87**	**0.13**	0.39	0.61	0.71
GOTA	T	0.48	0.79	0.29	0.49	**0.23**	0.86	**0.13**	0.38	0.58	0.69
GOCat	T+A	0.49	0.78	0.28	0.49	0.21	0.85	0.12	0.34	0.56	0.65
GOCat	T	0.50	0.76	0.27	0.49	0.21	0.83	0.12	0.34	0.52	0.63
RandFR	N/A	0.14	0.46	0.15	0.14	0.14	0.51	0.08	0.01	0.08	0.13
RandIC	N/A	0.28	0.34	0.23	0.26	0.11	0.46	0.06	0.00	0.00	0.00

Tab. 45: Statistical significance (p-value) of the paired t-test between GOTA (PM) and the other methods over BP (323 publications)

Method[a]	Info[b]	iP_1	iR_{20}	iP_{20}	hR_{20}	hP_{20}	MRR_{20}	R_{20}	$\#_{20}^{d}$
GOTA	T+A	0.01154	**0.1022**	**0.1432**	**0.3368**	**0.9155**	0.0005278	0.02312	**0.06341**
GOTA	T	0.003125	0.0016	0.0001272	0.0396	0.0005695	0.0001855	0.0001331	0.005187
GOCat	T+A	**0.1187**	0.02624	0.002076	0.02464	0.004969	0.0002339	0.0002465	0.001439
GOCat	T	**0.2011**	6.893e-05	1.083e-06	5.473e-05	1.067e-07	1.018e-05	1.828e-07	1.528e-05
RandFR	N/A	<2.2e-16	<2.2e-16	<2.2e-16	<2.2e-16	<2.2e-16	<2.2e-16	<2.2e-16	<2.2e-16
RandIC	N/A	<2.2e-16	<2.2e-16	4.533e-15	<2.2e-16	<2.2e-16	<2.2e-16	<2.2e-16	<2.2e-16

Not significant p-values are highlighted in bold.

Tab. 46: Performance comparison over MF (138 publications)

Method[a]	Info[b]	IT[c]			CAFA[c]	BC[c]			TREC[c]		$\#_{20}^{d}$
		iP_1	iR_{20}	iP_{20}	hF_{max}	hF_{20}	hR_{20}	hP_{20}	MRR_{20}	R_{20}	
GOTA	PM	**0.68**	**0.87**	**0.35**	**0.71**	0.29	**0.92**	0.17	**0.59**	**0.80**	**0.83**
GOTA	T+A	0.59	0.86	0.34	0.67	0.29	0.91	0.17	0.51	0.76	0.80
GOTA	T	0.58	0.86	0.34	0.66	0.29	0.91	0.17	0.51	0.75	0.78
GOCat	T+A	0.54	0.82	0.31	0.63	0.27	0.87	0.16	0.42	0.66	0.67
GOCat	T	0.51	0.82	0.31	0.62	0.26	0.87	0.15	0.42	0.65	0.67
RandFR	N/A	0.25	0.41	0.22	0.31	0.15	0.47	0.09	0.07	0.13	0.16
RandIC	N/A	0.25	0.24	0.25	0.31	**0.31**	0.23	**0.48**	0.04	0.03	0.04

Tab. 47: Statistical significance (p-value) of the paired t-test between GOTA (PM) and the other methods over MF (138 publications)

Method[a]	Info[b]	iP_1	iR_{20}	iP_{20}	hR_{20}	hP_{20}	MRR_{20}	R_{20}	$\#^d_{20}$
GOTA	T+A	0.0001881	**0.3638**	0.02657	**0.1034**	**0.4848**	1.067e-05	0.00555	0.02255
GOTA	T	0.0001762	**0.2592**	**0.1702**	**0.1080**	**0.1992**	6.814e-06	0.004927	0.006883
GOCat	T+A	8.935e-05	0.01198	0.0002518	0.008805	0.01927	9.73e-07	4.018e-05	7.772e-06
GOCat	T	1.639e-06	0.004354	3.987e-06	0.001591	0.01261	1.247e-07	1.752e-05	1.686e-05
RandFR	N/A	<2.2e-16	<2.2e-16	<2.2e-16	<2.2e-16	1.324e-14	<2.2e-16	<2.2e-16	<2.2e-16
RandIC	N/A	<2.2e-16	<2.2e-16	4.098e-07	<2.2e-16	**1**	<2.2e-16	<2.2e-16	<2.2e-16

Not significant p-values are highlighted in bold.

Tab. 48: Performance comparison over CC (83 publications)

Method[a]	Info[b]	IT[c]			CAFA[c]	BC[c]			TREC[c]		$\#^d_{20}$
		iP_1	iR_{20}	iP_{20}	hF_{max}	hF_{20}	hR_{20}	hP_{20}	MRR_{20}	R_{20}	
GOTA	PM	**0.58**	**0.88**	**0.42**	**0.64**	0.42	**0.91**	0.27	**0.44**	**0.71**	**0.73**
GOTA	T+A	0.56	**0.88**	0.41	0.62	0.40	**0.91**	0.26	0.43	0.69	0.71
GOTA	T	0.52	0.86	0.41	0.59	0.40	0.90	0.26	0.37	0.65	0.67
GOCat	T+A	0.50	0.82	0.38	0.60	0.38	0.88	0.24	0.37	0.66	0.70
GOCat	T	0.50	0.79	0.38	0.58	0.36	0.86	0.23	0.35	0.59	0.63
RandFR	N/A	0.41	0.65	0.31	0.47	0.26	0.73	0.16	0.17	0.40	0.43
RandIC	N/A	0.42	0.41	**0.42**	0.49	**0.49**	0.47	**0.51**	0.01	0.01	0.01

For each metric, the best result is highlighted in bold.

Tab. 49: Statistical significance (p-value) of the paired t-test between GOTA (PM) and the other methods over CC (83 publications)

Method[a]	Info[b]	iP_1	iR_{20}	iP_{20}	hR_{20}	hP_{20}	MRR_{20}	R_{20}	$\#^d_{20}$
GOTA	T+A	**0.2288**	**0.4065**	**0.2024**	**0.3352**	**0.3957**	**0.2664**	**0.3081**	**0.2088**
GOTA	T	0.0281	**0.1024**	**0.1016**	**0.1524**	**0.356**	0.01548	**0.1002**	**0.08345**
GOCat	T+A	0.02166	0.0253	0.003931	**0.09513**	0.04871	**0.07129**	**0.1931**	**0.2473**
GOCat	T	0.02317	0.001349	0.0002317	0.02060	0.005946	0.01124	0.01621	0.02446
RandFR	N/A	1.049e-05	1.837e-09	2.812e-14	6.79e-09	2.061e-10	3.845e-08	1.881e-06	4.42e-06
RandIC	N/A	3.247e-05	<2.2e-16	**0.5641**	<2.2e-16	**1**	1.735e-14	<2.2e-16	<2.2e-16

Not significant p-values are highlighted in bold.

[a] Method used for prediction. RandFR and RandIC are baseline predictors.

[b] Informations used in prediction: PM = title, abstract, references and publication year (PubMed); T+A = title and abstract; T = title; N/A = no information.

[c] Metrics definitions are in Section 2.3. For each metric, the best result is highlighted in bold.

[d] Fraction of publications for which at least one gold-standard annotation has been correctly predicted.

3 Testing authorship influence in classification performace

An interesting question is whether authorship can introduce some bias in the experimental testing. In particular, we ask to which extent having in test and training papers from the same author(s) can affect the classification performance. The authorship information can be useful for two reasons: i) the way in which an author writes can be repetitive in some parts and it could affect the text similarities extracted by an automatic classifier, and ii) it is conceivable that an author, or an authors list, could work and publish more than one papers on similar arguments and fields. In order to test the influence of authorship we perform two distinct experiments:

- We test GOTA's performances on the subset of test publications (319 over 15,000) for which there is not authorship overlap with the publications in the KB (Section 3.1)
- We compare the performances of GOTA with those of a simple k-NN approach that exploits uniquely the authorship information (Section 3.2)

The results of these tests are commented in the two following sections.

3.1 Subset of test-publications with no common author in the KB

In this section we analyze GOTA's performances over the subset of test publications (319 over 15,000) for which there is not authorship overlap with the publications in the KB. In these tests, GOTA's performances with full information (PM) are better in comparison to the other approaches, although the overall scores are slightly lower in comparison to GOTA's performances over the entire set of 15,000 publications (compare, for example, Table 50 with Table 2). The drop in performance accuracy can be observed for all the compared approaches, including the two naive classifiers RandIC and RandFR. The reason of such loss of accuracy is very likely related to the fact that the 319 publications considered in this test are mostly related to *hard* species. In fact, as an example, 39% of the 319 publications are related to Rat in comparison to 16% of the entire test set of 15,000 publications. As a further confirmation, by comparing Tables 50 and 18, we can see that GOTA's performances on the 319 publications are perfectly consistent with those on the Rat subset.

3.1.1 Evaluation of the top-10 predicted GO terms

Tab. 50: Performance comparison over all GO (319 publications)

Method[a]	Info[b]	IT[c]			CAFA[c]	BC[c]			TREC[c]		$\#_{10}^{d}$
		iP_1	iR_{10}	iP_{10}	hF_{max}	hF_{10}	hR_{10}	hP_{10}	MRR_{10}	R_{10}	
GOTA	PM	**0.39**	**0.61**	**0.22**	**0.40**	**0.26**	**0.67**	**0.16**	**0.36**	**0.43**	**0.57**
GOTA	T+A	**0.39**	**0.61**	0.21	0.39	**0.26**	**0.67**	**0.16**	0.35	0.42	0.55
GOTA	T	**0.39**	0.60	0.21	0.39	**0.26**	0.66	**0.16**	0.35	0.41	0.55
RandFR	N/A	0.12	0.27	0.10	0.15	0.11	0.30	0.07	0.09	0.10	0.15
RandIC	N/A	0.20	0.25	0.17	0.15	0.13	0.31	0.08	0.01	0.04	0.06
GOTA Φ_P	PM	0.32	0.60	0.20	0.39	**0.26**	0.65	**0.16**	0.33	0.41	0.53
GOTA Φ_P	T+A	0.31	0.60	0.20	0.37	0.24	0.64	0.15	0.31	0.39	0.53
GOTA Φ_P	T	0.31	0.60	0.20	0.37	0.24	0.64	0.15	0.31	0.39	0.53
GOTA Φ_T	PM	0.20	0.41	0.17	0.27	0.19	0.50	0.12	0.13	0.20	0.28
GOTA Φ_T	T+A	0.17	0.36	0.16	0.22	0.18	0.45	0.11	0.08	0.15	0.20
GOTA Φ_T	T	0.18	0.34	0.15	0.22	0.16	0.43	0.10	0.08	0.13	0.18

For each metric, the best result is highlighted in bold.

Tab. 51: Performance comparison over BP (234 publications)

Method[a]	Info[b]	IT[c]			CAFA[c]	BC[c]			TREC[c]		$\#_{10}^{d}$
		iP_1	iR_{10}	iP_{10}	hF_{max}	hF_{10}	hR_{10}	hP_{10}	MRR_{10}	R_{10}	
GOTA	PM	0.43	**0.66**	**0.27**	0.46	**0.26**	**0.72**	**0.16**	0.33	**0.43**	**0.50**
GOTA	T+A	0.42	0.65	**0.27**	0.45	0.25	0.71	0.15	0.32	0.42	0.47
GOTA	T	0.42	0.65	**0.27**	0.44	0.25	0.71	0.15	0.32	**0.43**	0.49
RandFR	N/A	0.18	0.34	0.17	0.13	0.12	0.33	0.07	0.03	0.04	0.07
RandIC	N/A	0.27	0.32	0.25	0.22	0.16	0.36	0.10	0.01	0.01	0.01
GOTA Φ_P	PM	**0.45**	**0.66**	**0.27**	**0.47**	0.23	0.71	0.14	**0.34**	**0.43**	0.49
GOTA Φ_P	T+A	0.42	0.65	**0.27**	0.45	0.25	0.70	0.15	0.32	0.41	0.48
GOTA Φ_P	T	0.42	0.65	**0.27**	0.44	0.25	0.70	0.15	0.32	0.41	0.48
GOTA Φ_T	PM	0.25	0.47	0.20	0.32	0.21	0.57	0.13	0.11	0.19	0.24
GOTA Φ_T	T+A	0.23	0.44	0.19	0.30	0.20	0.54	0.12	0.08	0.14	0.17
GOTA Φ_T	T	0.23	0.43	0.19	0.29	0.20	0.53	0.12	0.08	0.13	0.16

For each metric, the best result is highlighted in bold.

Tab. 52: Performance comparison over MF (109 publications)

Method[a]	Info[b]	IT[c]			CAFA[c]	BC[c]			TREC[c]		$\#^d_{10}$
		iP_1	iR_{10}	iP_{10}	hF_{max}	hF_{10}	hR_{10}	hP_{10}	MRR_{10}	R_{10}	
GOTA	PM	**0.65**	**0.72**	**0.32**	**0.70**	0.28	**0.85**	0.17	**0.52**	**0.62**	**0.69**
GOTA	T+A	0.62	**0.72**	**0.32**	0.69	0.28	**0.85**	0.17	0.50	0.59	0.65
GOTA	T	0.63	0.71	**0.32**	0.69	0.28	0.84	0.17	0.50	0.59	0.65
RandFR	N/A	0.36	0.41	0.24	0.46	0.14	0.55	0.08	0.18	0.20	0.22
RandIC	N/A	0.36	0.43	0.28	0.46	**0.33**	0.49	**0.25**	0.17	0.19	0.20
GOTA Φ_P	PM	0.58	0.71	0.31	0.68	0.27	0.84	0.16	0.47	0.59	0.65
GOTA Φ_P	T+A	0.56	0.70	0.30	0.66	0.27	0.83	0.16	0.43	0.54	0.60
GOTA Φ_P	T	0.56	0.70	0.30	0.66	0.27	0.83	0.16	0.43	0.54	0.60
GOTA Φ_T	PM	0.36	0.54	0.29	0.47	0.30	0.68	0.19	0.23	0.33	0.37
GOTA Φ_T	T+A	0.32	0.45	0.28	0.45	0.28	0.60	0.18	0.15	0.19	0.22
GOTA Φ_T	T	0.30	0.45	0.28	0.44	0.29	0.61	0.19	0.14	0.19	0.22

For each metric, the best result is highlighted in bold.

Tab. 53: Performance comparison over CC (88 publications)

Method[a]	Info[b]	IT[c]			CAFA[c]	BC[c]			TREC[c]		$\#^d_{10}$
		iP_1	iR_{10}	iP_{10}	hF_{max}	hF_{10}	hR_{10}	hP_{10}	MRR_{10}	R_{10}	
GOTA	PM	0.58	0.85	0.40	0.59	0.34	0.88	0.21	**0.50**	0.74	0.81
GOTA	T+A	**0.59**	0.86	0.40	**0.60**	0.33	0.89	0.20	**0.50**	0.75	0.81
GOTA	T	0.57	0.86	0.40	0.58	0.33	0.89	0.20	0.48	0.75	0.81
RandFR	N/A	0.45	0.71	0.36	0.45	0.34	0.73	0.22	0.25	0.54	0.61
RandIC	N/A	0.45	0.50	**0.42**	0.46	**0.42**	0.55	**0.34**	0.11	0.20	0.26
GOTA Φ_P	PM	0.54	0.85	0.39	0.57	0.34	0.88	0.21	0.46	0.73	0.82
GOTA Φ_P	T+A	0.55	**0.88**	0.40	0.56	0.34	**0.90**	0.21	0.47	**0.78**	**0.84**
GOTA Φ_P	T	0.55	0.87	0.40	0.56	0.34	**0.90**	0.21	0.46	0.76	0.83
GOTA Φ_T	PM	0.36	0.41	0.29	0.46	0.32	0.53	0.23	0.16	0.18	0.22
GOTA Φ_T	T+A	0.34	0.42	0.28	0.44	0.32	0.52	0.23	0.13	0.18	0.19
GOTA Φ_T	T	0.32	0.36	0.25	0.42	0.29	0.48	0.21	0.08	0.11	0.12

For each metric, the best result is highlighted in bold.

[a] Method used for prediction. RandFR and RandIC are baseline predictors.

[b] Informations used in prediction: PM = title, abstract, references and publication year (PubMed); T+A = title and abstract; T = title; N/A = no information.

[c] Metrics definitions are in Section 2.3.

[d] Fraction of publications for which at least one gold-standard annotation has been correctly predicted.

3.1.2 Evaluation of the top-20 predicted GO terms

Tab. 54: Performance comparison over all GO (319 publications)

Method[a]	Info[b]	IT[c]			CAFA[c]	BC[c]			TREC[c]		$\#^d_{20}$
		iP_1	iR_{20}	iP_{20}	hF_{max}	hF_{20}	hR_{20}	hP_{20}	MRR_{20}	R_{20}	
GOTA	PM	**0.39**	0.69	**0.19**	**0.40**	**0.19**	0.75	**0.11**	**0.37**	0.50	**0.63**
GOTA	T+A	**0.39**	**0.70**	**0.19**	0.39	0.18	**0.76**	0.10	0.35	**0.51**	**0.63**
GOTA	T	**0.39**	0.69	0.18	0.39	0.18	**0.76**	0.10	0.35	**0.51**	**0.63**
RandFR	N/A	0.12	0.39	0.11	0.15	0.10	0.41	0.06	0.09	0.14	0.24
RandIC	N/A	0.20	0.29	0.17	0.15	0.07	0.37	0.04	0.01	0.04	0.06
GOTA Φ_P	PM	0.32	0.69	0.17	0.39	0.18	0.75	0.10	0.33	0.50	**0.63**
GOTA Φ_P	T+A	0.31	0.68	0.17	0.37	0.18	0.74	0.10	0.31	0.48	0.61
GOTA Φ_P	T	0.31	0.68	0.17	0.37	0.18	0.74	0.10	0.31	0.48	0.61
GOTA Φ_T	PM	0.20	0.48	0.16	0.26	0.14	0.57	0.08	0.13	0.25	0.35
GOTA Φ_T	T+A	0.17	0.44	0.15	0.22	0.14	0.54	0.08	0.09	0.20	0.26
GOTA Φ_T	T	0.18	0.42	0.14	0.22	0.14	0.52	0.08	0.08	0.17	0.22

For each metric, the best result is highlighted in bold.

Tab. 55: Performance comparison over BP (234 publications)

Method[a]	Info[b]	IT[c]			CAFA[c]	BC[c]			TREC[c]		$\#^d_{20}$
		iP_1	iR_{20}	iP_{20}	hF_{max}	hF_{20}	hR_{20}	hP_{20}	MRR_{20}	R_{20}	
GOTA	PM	0.43	**0.74**	**0.24**	0.46	**0.18**	**0.79**	**0.10**	0.33	**0.50**	**0.56**
GOTA	T+A	0.42	0.73	0.23	0.45	**0.18**	0.78	**0.10**	0.32	0.49	0.55
GOTA	T	0.42	0.72	0.23	0.44	**0.18**	0.78	**0.10**	0.32	0.49	0.54
RandFR	N/A	0.18	0.44	0.15	0.13	0.11	0.46	0.06	0.03	0.09	0.14
RandIC	N/A	0.27	0.36	**0.24**	0.22	0.09	0.46	0.05	0.01	0.01	0.01
GOTA Φ_P	PM	**0.45**	**0.74**	0.23	**0.47**	**0.18**	**0.79**	**0.10**	**0.34**	0.49	0.55
GOTA Φ_P	T+A	0.42	0.72	0.23	0.45	0.16	0.77	0.09	0.32	0.47	0.54
GOTA Φ_P	T	0.42	0.72	0.23	0.44	0.16	0.77	0.09	0.32	0.47	0.54
GOTA Φ_T	PM	0.25	0.54	0.19	0.32	0.17	0.64	**0.10**	0.11	0.25	0.29
GOTA Φ_T	T+A	0.23	0.51	0.18	0.30	0.16	0.61	0.09	0.08	0.21	0.24
GOTA Φ_T	T	0.23	0.49	0.18	0.29	0.16	0.60	0.09	0.09	0.18	0.21

For each metric, the best result is highlighted in bold.

Tab. 56: Performance comparison over MF (109 publications)

Method[a]	Info[b]	IT[c]			CAFA[c]	BC[c]			TREC[c]		$\#^{d}_{20}$
		iP_1	iR_{20}	iP_{20}	hF_{max}	hF_{20}	hR_{20}	hP_{20}	MRR_{20}	R_{20}	
GOTA	PM	**0.65**	**0.76**	**0.29**	**0.70**	0.18	**0.89**	0.10	**0.52**	**0.67**	**0.73**
GOTA	T+A	0.62	**0.76**	**0.29**	0.69	0.18	**0.89**	0.10	0.50	0.66	0.72
GOTA	T	0.63	**0.76**	0.28	0.69	0.18	0.88	0.10	0.50	0.66	0.72
RandFR	N/A	0.36	0.46	0.23	0.46	0.11	0.58	0.06	0.18	0.21	0.24
RandIC	N/A	0.36	0.47	0.26	0.46	0.22	0.54	0.14	0.17	0.20	0.22
GOTA Φ_P	PM	0.58	0.74	0.27	0.68	0.18	0.88	0.10	0.47	**0.67**	0.72
GOTA Φ_P	T+A	0.56	0.75	0.27	0.66	0.18	0.88	0.10	0.44	0.64	0.71
GOTA Φ_P	T	0.56	0.75	0.27	0.66	0.18	0.88	0.10	0.44	0.64	0.71
GOTA Φ_T	PM	0.36	0.56	0.28	0.47	**0.26**	0.70	**0.16**	0.23	0.35	0.39
GOTA Φ_T	T+A	0.32	0.48	0.28	0.45	**0.26**	0.63	**0.16**	0.16	0.25	0.28
GOTA Φ_T	T	0.30	0.49	0.28	0.44	**0.26**	0.64	**0.16**	0.14	0.23	0.28

For each metric, the best result is highlighted in bold.

Tab. 57: Performance comparison over CC (88 publications)

Method[a]	Info[b]	IT[c]			CAFA[c]	BC[c]			TREC[c]		$\#^{d}_{20}$
		iP_1	iR_{20}	iP_{20}	hF_{max}	hF_{20}	hR_{20}	hP_{20}	MRR_{20}	R_{20}	
GOTA	PM	0.58	**0.90**	0.35	0.59	0.23	**0.93**	0.13	**0.51**	0.81	0.88
GOTA	T+A	**0.59**	0.89	0.35	**0.60**	0.23	0.92	0.13	0.50	0.82	0.88
GOTA	T	0.57	0.89	0.35	0.58	0.23	0.92	0.13	0.49	0.82	0.88
RandFR	N/A	0.45	0.81	0.33	0.45	0.23	0.84	0.13	0.26	0.63	0.73
RandIC	N/A	0.45	0.58	**0.37**	0.46	0.26	0.64	0.16	0.11	0.29	0.35
GOTA Φ_P	PM	0.54	**0.90**	0.35	0.57	0.23	**0.93**	0.13	0.47	**0.83**	**0.89**
GOTA Φ_P	T+A	0.55	0.89	0.35	0.56	0.23	**0.93**	0.13	0.47	0.82	0.88
GOTA Φ_P	T	0.55	0.89	0.35	0.56	0.23	**0.93**	0.13	0.47	0.82	0.88
GOTA Φ_T	PM	0.36	0.42	0.29	0.46	**0.32**	0.53	**0.23**	0.16	0.19	0.23
GOTA Φ_T	T+A	0.34	0.42	0.28	0.44	**0.32**	0.53	**0.23**	0.13	0.19	0.20
GOTA Φ_T	T	0.32	0.35	0.25	0.42	0.28	0.48	0.20	0.08	0.11	0.12

For each metric, the best result is highlighted in bold.

[a] Method used for prediction. RandFR and RandIC are baseline predictors.

[b] Informations used in prediction: PM = title, abstract, references and publication year (PubMed); T+A = title and abstract; T = title; N/A = no information.

[c] Metrics definitions are in Section 2.3.

[d] Fraction of publications for which at least one gold-standard annotation has been correctly predicted.

3.2 Subset of test-publications with common author in the KB

In this section we investigate the prediction capabilities associated to the authorship information. We consider uniquely the subset of test publications (14,681 over 15,000) for which there is some authorship overlap with the publications in the KB. We implemented a new k-NN baseline classifier that simply exploits the amount of overlap between the author names associated to two publications. The author-based k-NN approach (indicated as k-NN in the following tables) is quite similar to the approach implemented for the similarity score Φ_P in Eq. 2 of the paper:

1. we define the similarity score between two publications as the fraction of their common authors;
2. given a query publication, we use the author-based similarity score to select from the KB the top-150 most similar documents;
3. we transfer the weighted annotations from the 150 selected publications to the query.

We further include in the comparison a modified version of the original Φ_P score (which we indicate as GOTA Φ^*_P in the following tables): the k-NN approach that implements the Φ_P score has been modified in order to simply ignore publications in the KB when it detects some authorship overlap with the query. In practice, the GOTA Φ^*_P classifiers makes use of all the information in the KB that is ignored by the author-based k-NN classifier (and conversely).

In the following tables we can notice that the performances of the author-based k-NN approach are significantly better than those of the two random classifiers RandIC and RandFR. On the other end, they are still quite low in comparison to GOTA's capabilities. We can also notice that the k-NN approach GOTA Φ^*_P performs overall better than author-based k-NN classifier. These results suggest that, although authorship information can indirectly introduce some favorable bias in the classification, this is not the strongest source of information for GOTA. A further confirmation of this fact comes from the comparison between GOTA Φ^*_P and GOTA Φ_P performances, which are almost indistinguishable.

3.2.1 Evaluation of the top-10 predicted GO terms

Tab. 58: Performance comparison over all GO (14,681 publications)

Method[a]	Info[b]	IT[c]			CAFA[c]	BC[c]			TREC[c]		$\#_{10}^{d}$
		iP_1	iR_{10}	iP_{10}	hF_{max}	hF_{10}	hR_{10}	hP_{10}	MRR_{10}	R_{10}	
GOTA	PM	**0.43**	**0.64**	**0.28**	**0.43**	**0.35**	**0.69**	**0.23**	**0.40**	**0.46**	**0.69**
GOTA	T+A	0.42	**0.64**	**0.28**	**0.43**	0.34	0.68	**0.23**	0.39	0.45	0.67
GOTA	T	0.41	0.63	**0.28**	0.42	0.34	0.68	**0.23**	0.39	0.44	0.67
RandFR	N/A	0.20	0.33	0.15	0.20	0.17	0.33	0.11	0.18	0.15	0.28
RandIC	N/A	0.21	0.27	0.20	0.18	0.17	0.31	0.12	0.03	0.08	0.15
k-NN	AN	0.29	0.50	0.21	0.33	0.26	0.54	0.17	0.26	0.26	0.44
GOTA Φ_P^*	PM	0.36	0.62	0.25	0.40	0.33	0.66	0.22	0.37	0.41	0.63
GOTA Φ_P	PM	0.37	**0.64**	0.26	0.41	0.34	0.67	**0.23**	0.38	0.44	0.66
GOTA Φ_P	T+A	0.36	0.62	0.25	0.40	0.33	0.66	0.22	0.36	0.41	0.64
GOTA Φ_P	T	0.36	0.62	0.25	0.40	0.33	0.66	0.22	0.36	0.41	0.64
GOTA Φ_T	PM	0.28	0.41	0.22	0.30	0.26	0.49	0.18	0.16	0.17	0.32
GOTA Φ_T	T+A	0.24	0.37	0.20	0.27	0.24	0.46	0.16	0.11	0.12	0.23
GOTA Φ_T	T	0.23	0.35	0.19	0.26	0.22	0.44	0.15	0.09	0.10	0.19

For each metric, the best result is highlighted in bold.

Tab. 59: Performance comparison over BP (11,008 publications)

Method[a]	Info[b]	IT[c]			CAFA[c]	BC[c]			TREC[c]		$\#_{10}^{d}$
		iP_1	iR_{10}	iP_{10}	hF_{max}	hF_{10}	hR_{10}	hP_{10}	MRR_{10}	R_{10}	
GOTA	PM	**0.50**	**0.72**	**0.34**	**0.50**	**0.34**	**0.76**	**0.22**	**0.38**	**0.49**	**0.64**
GOTA	T+A	0.49	0.71	**0.34**	0.49	**0.34**	0.75	**0.22**	0.37	0.47	0.62
GOTA	T	0.49	0.70	**0.34**	0.49	**0.34**	0.75	**0.22**	0.36	0.47	0.62
RandFR	N/A	0.21	0.35	0.20	0.17	0.16	0.35	0.10	0.04	0.06	0.10
RandIC	N/A	0.29	0.32	0.26	0.26	0.20	0.36	0.14	0.01	0.01	0.03
k-NN	AN	0.35	0.55	0.25	0.37	0.26	0.60	0.17	0.19	0.23	0.32
GOTA Φ_P^*	PM	0.47	0.69	0.32	0.47	0.33	0.74	0.21	0.33	0.44	0.58
GOTA Φ_P	PM	0.48	0.71	0.33	0.48	0.33	0.75	0.21	0.36	0.46	0.61
GOTA Φ_P	T+A	0.47	0.69	0.33	0.47	0.33	0.74	0.21	0.33	0.44	0.59
GOTA Φ_P	T	0.47	0.69	0.33	0.47	0.33	0.74	0.21	0.33	0.44	0.59
GOTA Φ_T	PM	0.33	0.51	0.27	0.37	0.29	0.59	0.19	0.15	0.21	0.31
GOTA Φ_T	T+A	0.29	0.47	0.25	0.34	0.27	0.56	0.18	0.11	0.15	0.22
GOTA Φ_T	T	0.27	0.45	0.24	0.32	0.26	0.54	0.17	0.09	0.13	0.19

For each metric, the best result is highlighted in bold.

Tab. 60: Performance comparison over MF (6,910 publications)

Method[a]	Info[b]	IT[c]			CAFA[c]	BC[c]			TREC[c]		$\#_{10}^{d}$
		iP_1	iR_{10}	iP_{10}	hF_{max}	hF_{10}	hR_{10}	hP_{10}	MRR_{10}	R_{10}	
GOTA	PM	**0.60**	**0.75**	**0.35**	**0.65**	0.33	**0.88**	0.20	**0.54**	**0.71**	**0.79**
GOTA	T+A	0.57	0.74	**0.35**	0.64	0.33	**0.88**	0.20	0.52	0.70	0.78
GOTA	T	0.57	0.74	**0.35**	0.64	0.33	0.87	0.20	0.52	0.69	0.77
RandFR	N/A	0.43	0.46	0.27	0.49	0.17	0.59	0.10	0.31	0.32	0.40
RandIC	N/A	0.43	0.45	0.32	0.49	**0.35**	0.50	**0.27**	0.29	0.26	0.32
k-NN	AN	0.46	0.57	0.29	0.54	0.29	0.70	0.18	0.37	0.43	0.52
GOTA Φ_P^*	PM	0.54	0.73	0.34	0.64	0.31	0.86	0.19	0.50	0.66	0.74
GOTA Φ_P	PM	0.55	**0.75**	0.34	**0.65**	0.31	**0.88**	0.19	0.52	0.69	0.77
GOTA Φ_P	T+A	0.53	0.74	0.34	0.62	0.31	0.87	0.19	0.49	0.67	0.75
GOTA Φ_P	T	0.53	0.74	0.34	0.63	0.31	0.87	0.19	0.49	0.67	0.75
GOTA Φ_T	PM	0.36	0.50	0.29	0.48	0.32	0.66	0.21	0.17	0.25	0.30
GOTA Φ_T	T+A	0.32	0.45	0.27	0.44	0.30	0.62	0.20	0.12	0.18	0.23
GOTA Φ_T	T	0.30	0.44	0.27	0.43	0.29	0.61	0.19	0.10	0.17	0.21

For each metric, the best result is highlighted in bold.

Tab. 61: Performance comparison over CC (5,114 publications)

Method[a]	Info[b]	IT[c]			CAFA[c]	BC[c]			TREC[c]		$\#^{d}_{10}$
		iP_1	iR_{10}	iP_{10}	hF_{max}	hF_{10}	hR_{10}	hP_{10}	MRR_{10}	R_{10}	
GOTA	PM	**0.62**	**0.86**	0.43	**0.64**	0.40	**0.90**	0.26	**0.52**	**0.73**	**0.81**
GOTA	T+A	0.61	0.85	0.42	**0.64**	0.39	**0.90**	0.25	0.51	0.71	0.80
GOTA	T	0.61	0.85	0.42	0.63	0.39	**0.90**	0.25	0.51	0.71	0.80
RandFR	N/A	0.50	0.68	0.37	0.52	0.38	0.71	0.26	0.30	0.48	0.56
RandIC	N/A	0.50	0.56	**0.46**	0.53	**0.50**	0.60	**0.43**	0.12	0.23	0.30
k-NN	AN	0.52	0.71	0.39	0.57	0.40	0.77	0.27	0.37	0.48	0.58
GOTA Φ^*_P	PM	0.60	0.85	0.42	0.63	0.40	0.89	0.26	0.50	0.70	0.78
GOTA Φ_P	PM	**0.62**	**0.86**	0.42	**0.64**	0.40	**0.90**	0.26	0.51	0.72	0.80
GOTA Φ_P	T+A	0.60	**0.86**	0.42	0.62	0.40	0.89	0.26	0.50	0.71	0.79
GOTA Φ_P	T	0.60	**0.86**	0.42	0.62	0.40	0.89	0.26	0.50	0.71	0.79
GOTA Φ_T	PM	0.35	0.43	0.29	0.45	0.34	0.55	0.25	0.14	0.19	0.24
GOTA Φ_T	T+A	0.32	0.41	0.28	0.43	0.34	0.53	0.25	0.11	0.16	0.21
GOTA Φ_T	T	0.31	0.37	0.27	0.42	0.32	0.51	0.23	0.09	0.12	0.16

For each metric, the best result is highlighted in bold.

[a] Method used for prediction. RandFR and RandIC are baseline predictors. k-NN is a baseline predictor that exploits uniquely authorship information. GOTA Φ^*_P is a modified version of GOTA Φ_P that ignores authorship information.

[b] Informations used in prediction: PM = title, abstract, references and publication year (PubMed); T+A = title and abstract; T = title; AN = author names; N/A = no information.

[c] Metrics definitions are in Section 2.3.

[d] Fraction of publications for which at least one gold-standard annotation has been correctly predicted.

3.2.2 Evaluation of the top-20 predicted GO terms

Tab. 62: Performance comparison over all GO (14681 publications)

Method[a]	Info[b]	IT[c]			CAFA[c]	BC[c]			TREC[c]		$\#^{d}_{20}$
		iP_1	iR_{20}	iP_{20}	hF_{max}	hF_{20}	hR_{20}	hP_{20}	MRR_{20}	R_{20}	
GOTA	PM	**0.43**	**0.73**	**0.24**	**0.43**	**0.27**	**0.80**	**0.16**	**0.41**	**0.57**	**0.77**
GOTA	T+A	0.42	0.72	**0.24**	**0.43**	**0.27**	0.79	**0.16**	0.40	0.55	0.76
GOTA	T	0.41	0.72	**0.24**	0.42	**0.27**	0.78	**0.16**	0.40	0.54	0.75
RandFR	N/A	0.20	0.40	0.14	0.20	0.15	0.43	0.09	0.18	0.19	0.35
RandIC	N/A	0.21	0.31	0.19	0.18	0.10	0.38	0.06	0.03	0.08	0.15
k-NN	AN	0.29	0.57	0.19	0.33	0.22	0.63	0.13	0.26	0.31	0.50
GOTA Φ^*_P	PM	0.36	0.71	0.22	0.40	0.25	0.77	0.15	0.37	0.52	0.73
GOTA Φ_P	PM	0.37	**0.73**	0.23	0.41	**0.27**	0.78	**0.16**	0.39	0.54	0.75
GOTA Φ_P	T+A	0.36	0.71	0.23	0.40	0.25	0.77	0.15	0.37	0.52	0.73
GOTA Φ_P	T	0.36	0.71	0.23	0.40	0.25	0.77	0.15	0.37	0.52	0.73
GOTA Φ_T	PM	0.28	0.48	0.20	0.31	0.21	0.59	0.13	0.16	0.22	0.40
GOTA Φ_T	T+A	0.24	0.45	0.19	0.27	0.20	0.56	0.12	0.11	0.16	0.31
GOTA Φ_T	T	0.23	0.42	0.19	0.26	0.20	0.54	0.12	0.09	0.14	0.27

For each metric, the best result is highlighted in bold.

Tab. 63: Performance comparison over BP (11,008 publications)

Method[a]	Info[b]	IT[c]			CAFA[c]	BC[c]			TREC[c]		$\#^{d}_{20}$
		iP_1	iR_{20}	iP_{20}	hF_{max}	hF_{20}	hR_{20}	hP_{20}	MRR_{20}	R_{20}	
GOTA	PM	**0.50**	**0.78**	**0.30**	**0.50**	**0.25**	**0.84**	**0.15**	**0.39**	**0.58**	**0.72**
GOTA	T+A	0.49	**0.78**	**0.30**	0.49	**0.25**	0.83	**0.15**	0.37	0.56	0.71
GOTA	T	0.49	0.77	**0.30**	0.49	0.24	0.83	0.14	0.37	0.56	0.70
RandFR	N/A	0.21	0.43	0.17	0.17	0.15	0.48	0.09	0.04	0.10	0.15
RandIC	N/A	0.29	0.36	0.25	0.26	0.14	0.47	0.08	0.01	0.02	0.03
k-NN	AN	0.35	0.61	0.23	0.37	0.22	0.67	0.13	0.19	0.27	0.37
GOTA Φ^*_P	PM	0.47	0.77	0.28	0.47	0.24	0.82	0.14	0.34	0.52	0.67
GOTA Φ_P	PM	0.48	**0.78**	0.28	0.48	0.24	0.83	0.14	0.36	0.55	0.70
GOTA Φ_P	T+A	0.47	0.77	0.28	0.47	0.24	0.82	0.14	0.34	0.53	0.67
GOTA Φ_P	T	0.47	0.77	0.28	0.47	0.24	0.82	0.14	0.34	0.53	0.67
GOTA Φ_T	PM	0.33	0.58	0.25	0.37	0.23	0.66	0.14	0.16	0.27	0.38
GOTA Φ_T	T+A	0.29	0.54	0.23	0.34	0.23	0.63	0.14	0.11	0.20	0.30
GOTA Φ_T	T	0.27	0.51	0.23	0.32	0.21	0.62	0.13	0.09	0.17	0.26

For each metric, the best result is highlighted in bold.

Tab. 64: Performance comparison over MF (6,910 publications)

Method[a]	Info[b]	IT[c]			CAFA[c]	BC[c]			TREC[c]		$\#^d_{20}$
		iP_1	iR_{20}	iP_{20}	hF_{max}	hF_{20}	hR_{20}	hP_{20}	MRR_{20}	R_{20}	
GOTA	PM	**0.60**	**0.78**	**0.31**	**0.65**	0.21	**0.92**	0.12	**0.54**	**0.77**	**0.84**
GOTA	T+A	0.57	0.77	0.30	0.64	0.21	**0.92**	0.12	0.53	0.76	0.83
GOTA	T	0.57	0.77	0.30	0.64	0.21	**0.92**	0.12	0.53	0.76	0.83
RandFR	N/A	0.43	0.53	0.26	0.49	0.13	0.64	0.07	0.31	0.38	0.45
RandIC	N/A	0.43	0.50	0.30	0.49	0.24	0.56	0.15	0.29	0.29	0.36
k-NN	AN	0.46	0.60	0.27	0.54	0.25	0.73	0.15	0.38	0.46	0.54
GOTA Φ^*_P	PM	0.54	0.77	0.29	0.64	0.20	0.91	0.11	0.50	0.73	0.80
GOTA Φ_P	PM	0.55	**0.78**	0.30	**0.65**	0.20	**0.92**	0.11	0.52	0.76	0.83
GOTA Φ_P	T+A	0.53	**0.78**	0.29	0.62	0.20	0.91	0.11	0.49	0.75	0.82
GOTA Φ_P	T	0.53	**0.78**	0.29	0.63	0.20	0.91	0.11	0.49	0.75	0.82
GOTA Φ_T	PM	0.36	0.52	0.28	0.48	**0.27**	0.69	**0.17**	0.17	0.28	0.34
GOTA Φ_T	T+A	0.32	0.48	0.26	0.44	**0.27**	0.64	**0.17**	0.12	0.22	0.27
GOTA Φ_T	T	0.30	0.46	0.26	0.43	0.26	0.63	0.16	0.11	0.20	0.25

For each metric, the best result is highlighted in bold.

Tab. 65: Performance comparison over CC (5,114 publications)

Method[a]	Info[b]	IT[c]			CAFA[c]	BC[c]			TREC[c]		$\#^d_{20}$
		iP_1	iR_{20}	iP_{20}	hF_{max}	hF_{20}	hR_{20}	hP_{20}	MRR_{20}	R_{20}	
GOTA	PM	**0.62**	**0.90**	0.38	**0.64**	0.27	**0.94**	0.16	**0.52**	**0.81**	**0.88**
GOTA	T+A	0.61	0.89	0.37	**0.64**	0.27	**0.94**	0.16	**0.52**	0.80	0.87
GOTA	T	0.61	0.89	0.37	0.63	0.27	**0.94**	0.16	0.51	0.79	0.86
RandFR	N/A	0.50	0.77	0.34	0.52	0.25	0.80	0.15	0.31	0.56	0.65
RandIC	N/A	0.50	0.66	**0.41**	0.53	0.32	0.71	0.21	0.13	0.38	0.46
k-NN	AN	0.52	0.75	0.37	0.57	**0.35**	0.80	0.22	0.37	0.53	0.62
GOTA Φ^*_P	PM	0.60	0.89	0.37	0.63	0.27	**0.94**	0.16	0.50	0.78	0.85
GOTA Φ_P	PM	**0.62**	**0.90**	0.37	**0.64**	0.27	**0.94**	0.16	**0.52**	0.80	0.87
GOTA Φ_P	T+A	0.60	**0.90**	0.37	0.62	0.27	**0.94**	0.16	0.50	0.79	0.86
GOTA Φ_P	T	0.60	**0.90**	0.37	0.62	0.27	**0.94**	0.16	0.50	0.79	0.86
GOTA Φ_T	PM	0.35	0.43	0.29	0.45	0.33	0.56	**0.23**	0.15	0.20	0.25
GOTA Φ_T	T+A	0.32	0.42	0.27	0.43	0.31	0.55	0.22	0.12	0.18	0.23
GOTA Φ_T	T	0.31	0.38	0.26	0.42	0.30	0.52	0.21	0.09	0.14	0.18

For each metric, the best result is highlighted in bold.

[a] Method used for prediction. RandFR and RandIC are baseline predictors. k-NN is a baseline predictor that exploits uniquely authorship information. GOTA Φ^*_P is a modified version of GOTA Φ_P that ignores authorship information.

[b] Informations used in prediction: PM = title, abstract, references and publication year (PubMed); T+A = title and abstract; T = title; AN = author names; N/A = no information.

[c] Metrics definitions are in Section 2.3.

[d] Fraction of publications for which at least one gold-standard annotation has been correctly predicted.

4 Confidence score

We considered the problem of assigning a confidence threshold to the predicted annotations. The aim of such filter is to provide to the user a confidence level, mainly for those queries that have a very low biological content. Such confidence scores are shown for a prediction when GOTA's Web-based application[1] is queried with some text or PubMed identifier.

We define three confidence levels: *low*, *medium*, *high*. We tuned a threshold value for each level of confidence by considering GOTA's score of the very top predicted term. In detail, we retrieved from Scopus[2] a set of 600 abstracts from six scientific fields not directly related to biology and medicine (namely: physics, chemistry, mathematics, computer science, economics and arts). We downloaded the top-100 cited publications in each discipline. We queried the 600 abstracts with GOTA and retrieved the score value of the very top predicted term for each publication. We then used such scores to setup the score thresholds for the *low*, *medium* and *high* confidence levels:

- we selected as *high* confidence threshold the score value that allows GOTA to *filter-out* 95% of the 600 query abstracts (i.e. for only 5% of the 600 test-abstracts the score assigned to the top predicted term is strictly higher than the *high* confidence threshold);
- we selected as medium confidence threshold the score value that allows GOTA to *filter-out* 90% of the 600 query abstracts.
- if for a query publication the top predicted term has a score strictly lower than the *medium* confidence threshold, then to such prediction is assigned a *low* confidence level.

Although this approach can be made more robust, it still offers some indications about the reliability of GOTA's classifications. In particular, we can roughly say that there is 5% probability that classifications of non biologically-related publications are reported as *highly confident*, and 90% probability that are reported as *low-confidence classifications*. These score thresholds seem to work quite well with the 15,000 biologically-related publications in our test set: 96% of the classifications are reported as high confident and 2% as low confident. Consistently, the average quality of the 2% low-confident classifications is also quite poor in comparison to GOTA's average performance.

[1] http://gota.apice.unibo.it
[2] http://www.scopus.com
